# Limits to high-speed simulations of spiking neural networks using general-purpose computers

**DOI:** 10.3389/fninf.2014.00076

**Published:** 2014-09-11

**Authors:** Friedemann Zenke, Wulfram Gerstner

**Affiliations:** School of Computer and Communication Sciences and Brain Mind Institute, School of Life Sciences, Ecole Polytechnique Fédérale de LausanneLausanne, Switzerland

**Keywords:** spiking neural networks, network simulator, synaptic plasticity, STDP, parallel computing, computational neuroscience

## Abstract

To understand how the central nervous system performs computations using recurrent neuronal circuitry, simulations have become an indispensable tool for theoretical neuroscience. To study neuronal circuits and their ability to self-organize, increasing attention has been directed toward synaptic plasticity. In particular spike-timing-dependent plasticity (STDP) creates specific demands for simulations of spiking neural networks. On the one hand a high temporal resolution is required to capture the millisecond timescale of typical STDP windows. On the other hand network simulations have to evolve over hours up to days, to capture the timescale of long-term plasticity. To do this efficiently, fast simulation speed is the crucial ingredient rather than large neuron numbers. Using different medium-sized network models consisting of several thousands of neurons and off-the-shelf hardware, we compare the simulation speed of the simulators: Brian, NEST and Neuron as well as our own simulator Auryn. Our results show that real-time simulations of different plastic network models are possible in parallel simulations in which numerical precision is not a primary concern. Even so, the speed-up margin of parallelism is limited and boosting simulation speeds beyond one tenth of real-time is difficult. By profiling simulation code we show that the run times of typical plastic network simulations encounter a hard boundary. This limit is partly due to latencies in the inter-process communications and thus cannot be overcome by increased parallelism. Overall, these results show that to study plasticity in medium-sized spiking neural networks, adequate simulation tools are readily available which run efficiently on small clusters. However, to run simulations substantially faster than real-time, special hardware is a prerequisite.

## 1. Introduction

Neurons communicate with each other by short electrical pulses, called action potentials or spikes, which can be considered as unitary events. In simple neuron models of integrate-and-fire type, such events are generated by a threshold crossing process. The dynamics of a single neuron, which forms one unit of a large brain network, are therefore relatively simple.

Nevertheless, the simulation of activity in large neural networks, which has been receiving increasing interest over the past years (Markram, 2006; Ananthanarayanan et al., 2009; Lang et al., 2011; Koch and Reid, 2012; Waldrop, 2012; Kandel et al., 2013), poses multiple computational challenges. First, brain networks consist of billions of neurons (Kandel et al., 2000). Even if each neuron is described as a relatively simple dynamic processing unit (e.g., an adaptive integrate-and-fire neuron with two or three update equations per neuron Izhikevich, 2003; Brette and Gerstner, 2005; Gerstner et al., 2014), the sheer number of units suggests that faster than real-time simulation of these equations will be hard to achieve on a single core. Hence parallelization of computation is desirable. Second, each unit sends and receives signals from thousands of others (DeFelipe and Fariñas, 1992; Kandel et al., 2000), such that connectivity between units is relatively high compared to classical models in the physical sciences where interactions are mainly between nearest neighbors in physical space (Anderson, 1995). Therefore, the communication overhead in a parallel implementation could potentially be high. Third, the synaptic contact points between two connected units are not fixed but may change (Bliss and Lømo, 1973; Markram et al., 1997; Bi and Poo, 1998; Zhang et al., 1998; Bi and Poo, 2001; Markram et al., 2012). Consequently, connections cannot be described with fixed parameters, but need further dynamic variables. Moreover, the evolution of these synaptic variables depends on activity of both the sending and the receiving neuron so that their treatment requires additional care and readily available parallelization approaches cannot be used. The changes in the dynamic values associated with the synaptic contact points are referred to as synaptic plasticity.

The question therefore arises whether the scaling of parallel implementations of simulated neural networks is dominated mainly by the inter-process communication or by the dynamics of the connections. This question cannot be answered in a straightforward manner, because it depends on multiple factors. First, the communication between neurons only takes place at the moment when a spike happens, leading to event-based updating schemes (Morrison et al., 2007, 2008). Accordingly, the number of events per unit of time plays a role for the communication load. Second, changes of synaptic parameters, while induced by spike events, are relatively small so that they evolve on a slower time scale. Roughly speaking, a biological neuron sends out spikes that last each about 1 ms. The rate at which these spike events are generated is a few per second. The slowest dynamics are those of synaptic plasticity which typically needs several spike events to induce a measurable change. Moreover, once changes are induced, they often persist for many hours. In the field of neuroscience, the behavioral phenomenon of learning and memory formation is thought to be intimately linked to the biological rules of synaptic plasticity (Bliss and Lømo, 1973; Markram et al., 1997; Bi and Poo, 1998; Zhang et al., 1998; Bi and Poo, 2001; Markram et al., 2012). To verify in experiments whether a stable memory has been formed it is not uncommon to follow a biological substrate for 24 h or more. If we want to simulate learning and memory formation, the simulation software has to cover time scales from milliseconds to days. To facilitate studies of learning and plasticity in network models it is therefore highly desirable to run simulations as fast as possible.

While simulation packages for networks with static (i.e., non-plastic) connections are readily available (Gewaltig and Diesmann, 2007; Eliasmith et al., 2012; Hoang et al., 2013), simulations of plastic brain circuits have received much less attention (Gewaltig and Diesmann, 2007; Izhikevich and Edelman, 2008; Ananthanarayanan et al., 2009). For example, the NEST simulation environment has been released initially for fixed network connections and models of synaptic plasticity have been added later on (Gewaltig and Diesmann, 2007; Morrison et al., 2007). Recently, increasing efforts are being made to enable real-time simulations by using specialized simulation hardware (Furber and Temple, 2007; Schemmel et al., 2010) or GPUs (Yudanov et al., 2010; Hoang et al., 2013).

Here we focus on networks of several thousands of neurons. These medium-sized networks are of particular practical importance because they are used in many theory and modeling labs worldwide. Since not all modeling labs have access to super computers we further limit our study to the use of general purpose computers, which can be used either individually or as clusters. In this framework we are interested in strong scaling, i.e., how fast a given network model of fixed size can be simulated.

To explore what is currently achievable using standard off-the-shelf hardware and publicly available software, we compare the results and execution times of three typical network simulations—with and without plasticity. In particular we use the multi-purpose simulation frameworks NEST, Neuron and Brian and compare them with our own simulator Auryn. Auryn has specifically been optimized to study plasticity in large-timescale simulations (up to days of simulated time). To minimize run times in such simulations Auryn uses forward Euler integration and relies on single precision arithmetic.

In this work we first analyze the trade-off between simulation precision and simulation speed. In particular we focus on a variant of the classic balanced network model by Vogels and Abbott (2005) and show that meaningful results can be obtained at high simulation speed when using numerical integration algorithms with a comparatively low fidelity (e.g., forward Euler method). We then turn to parallel simulations and analyze by how much network simulations can be sped up and how strong scaling is limited by multiple factors. In particular we identify inter-process communication and spike propagation as the two major limiting factors which prevent a further speed-up in simulations of medium-sized spiking neural networks.

In summary we show, by using three examples of standard balanced network models, that real-time simulations are well within reach with today's off-the-shelf hardware. However, the increase of simulation speed well beyond real-time, as required for studying synaptic plasticity and learning, calls for specialized hardware with low communication latencies.

## 2. Materials and methods

In this manuscript we compare results from a range of different neuron, network and plasticity models. However, there are some underlying similarities. All networks are built from integrate-and-fire neurons with either current based or conductance based synaptic input. We have summarized the detailed model description for the neuron models, plasticity rules and network models in tabular form according to Nordlie et al. (2009) (Supplementary Material). In the following we only give a short overview of the simulation code, hardware and network models we used. In Section 2.5 we comment on general implementation details of STDP in simulations.

### 2.1. Simulation code and hardware

For all simulations using Auryn (version 0.4), which is publicly available on the Internet^1^, we used forward Euler integration with a 0.1 ms integration time step and a 0.8 ms synaptic delay unless mentioned otherwise. Simulations were compiled against Boost (version 1.41.0) and MPICH2 (version 1.2.1) using the GNU C++ compiler (version 4.4.7). The code was executed on either a single node or a small cluster consisting of 4 nodes. The individual nodes were technically identical and running Red Hat Enterprise Linux (version 6) on a dual CPU (Intel Xeon CPU E5-2670 0 @ 2.60 GHz) board with 64 GB of RAM. Nodes communicated using Ethernet link aggregation over four 1 Gb connections each via a switch comprised of two Cisco Nexus Fabric Extenders (N2K-C2248TP) and two Cisco Nexus (N5K-C5548-UP). All *p*-values were computed from the two sample Kolmogorov-Smirnov test from the stats package in SciPy.

### 2.2. Simple run time model

To provide a simple model to describe the simulation run time *T* as measured in simulations with Auryn, we assume that

(1) $$T=T_{\text{sim}}+T_{\text{sync}}$$

where *T*_sim_ is the time spent on the actual simulation of neural variables and *T*_sync_ corresponds to the time spent on inter-process communications.

In an ideal parallel implementation *T*_sim_ scales as $\propto \frac{1}{n}$ where *n* is the number of cores that share the work. Auryn uses an AllGather operation to communicate spikes from one node to the other, which is generally implemented as either the Ring Algorithm, Recursive Doubling or the Bruck algorithm (Thakur et al., 2005). The respective algorithm is selected heuristically depending on message size and the number of nodes used for the simulation. While the Ring algorithm scales as $\propto \beta (n-1)+\gamma \frac{n-1}{n}$, the two other algorithms scale as $\propto \beta \left\lceil \log_{2}n\right\rceil +\gamma \frac{n-1}{n}$. Taken together we either expect the run time *T* to scale as

(2) $$T=\frac{\alpha }{n}+\beta \left\lceil \log_{2}n\right\rceil +\gamma \frac{n-1}{n}$$

or else

(3) $$T=\frac{\alpha }{n}+\beta (n-1)+\gamma \frac{n-1}{n}$$

with only positive parameters α, β, and γ. We can see immediately that both, Expression (2) and (3), are lower bounded: *T*_LB_ > 0. This is the manifestation of the plain fact that inter-process communication takes time and simulations cannot run faster than the time they spend on communication.

### 2.3. Network models

#### 2.3.1. Vogels-abbott benchmark

For comparison against other simulators we adapted the VAbenchmark2.py benchmark code from PyNN (Davison et al., 2009) which is a down-scaled version of a network by Vogels and Abbott (2005). The network has 3200 excitatory and 800 inhibitory neurons and approximately 320k synapses.

Specifically we re-implemented the same simulation in Auryn, Brian and NEST (Supplementary Material). The Auryn code is comprised as an example in the current release of Auryn (sim_coba_benchmark.cpp). For simulations in Neuron the original PyNN version (Davison et al., 2009) was modified. In particular all recordings were switched off and the script was used directly via nrniv -python -mpi to avoid unnecessary overhead.

We ensured that all simulations were loading the same weight matrix from external files. Unless mentioned otherwise we used a 0.8 ms synaptic delay between all synapses. The delay was chosen as a compromise between a small value, to stay as close as possible to the original (Vogels and Abbott, 2005), and a large value, to reduce inter-process communication for the cases where parallel execution was possible. Simulations were run for 60 s of simulated time. To avoid the spontaneous deactivation of the self-sustained activity state (Vogels and Abbott, 2005), all neurons received excitatory current input of 200 pA (Vogels et al., 2011). All benchmarks were run 5 times for statistics. For each simulation only the run time of the actual simulation was measured. The time to set up the weight matrices or to write data to disk was ignored.

To simulate this network we used Neuron (version 7.3; Carnevale and Hines, 2006), NEST (version 2.2.2; Gewaltig and Diesmann, 2007) and Brian (version 1.4.1; Goodman and Brette, 2008) and compiled against MPI libraries where possible. Unless mentioned otherwise we used MPICH2 (version 1.2.1). However, we did not encounter a notable difference in performance with OpenMPI (version 1.4.3).

#### 2.3.2. 25k cell network model

The plastic 25,000 cell network model was implemented as described in Zenke et al. (2013). It consists of 20,000 excitatory and 5000 inhibitory neurons connected by a total of ≈ 3.4×10^7^ synapses of which approximately ≈ 2×10^7^ are plastic as described by triplet STDP (Pfister and Gerstner, 2006). The code is available as sim_background.cpp (sim_bg_static.cpp for the non-plastic version of it) with the current release of the Auryn simulator. A detailed model description is available in tabular form in the Supplementary Material.

#### 2.3.3. Brunel network

This is a 10,000 cell balanced network model based on a network by Brunel (2000) with a total of 10^7^ synapses. Specifically we adapted the simulation included in the PyNEST examples in the current NEST (Gewaltig and Diesmann, 2007) release (version 2.2.2) under the LeNovere_2012 directory (Gewaltig et al., 2012), which comes as a non-plastic network and the same network with weight dependent STDP (Supplementary Material; 6.4×10^7^ plastic synapses). To make the networks comparable with the other simulations synaptic delays were set to 0.8 ms and the same random connectivity was loaded from external files. In contrast to Gewaltig et al. (2012) all weights were initialized with the same value to facilitate the implementation of a comparable simulation in Auryn.

### 2.4. The auryn simulator

Auryn was written with the UNIX philosophy in mind: Do one thing and do it well (Raymond, 2003). The Auryn code is open source and it was optimized for simulation speed to allow for large-time-scale simulations of recurrent neural networks involving synaptic plasticity. To that end Auryn simulates networks of spiking neurons and writes relevant output to human readable text files. It does not perform any analysis and the output files have to be processed and analyzed independently. At the heart Auryn is a collection of C++ classes that are combined into a compiled program to form the simulation. This allows the compiler to optimize each simulation code specifically for the hardware it runs on.

Like other simulators Auryn takes a hybrid approach between event-based and continuous integration (Morrison et al., 2005). Neuron models in Auryn are integrated continuously, while weight updates for many standard synaptic plasticity rules are implemented in an efficient event-based way.

Quantities that require time continuous integration are typically neuronal state variables describing synaptic conductance and membrane voltage. In many neuronal networks large sets of identical or similar neurons need to be integrated. The required computation can be vectorized efficiently. The advantages of vectorization are the reduction of function calls, the efficient use of layered cache architectures deployed in modern CPUs, and giving the compiler the opportunity to use hardware for single instruction multiple data (SIMD) such as SSE or AVX. Vectorization is therefore widely used in existing simulators such as Brian (Brette and Goodman, 2011) as well as in our code. Since many SIMD instructions can process twice the amount of single precision floating point operations per instruction, Auryn uses single precision arithmetic to increase performance.

The logical extension to vectorization is parallelization. To run parallel code Auryn uses the message passing interface (MPI) as a general and versatile back-end to allow parallel simulations on a single machine with multiple cores or distributed over multiple physical machines in a cluster.

**Figure 1:**
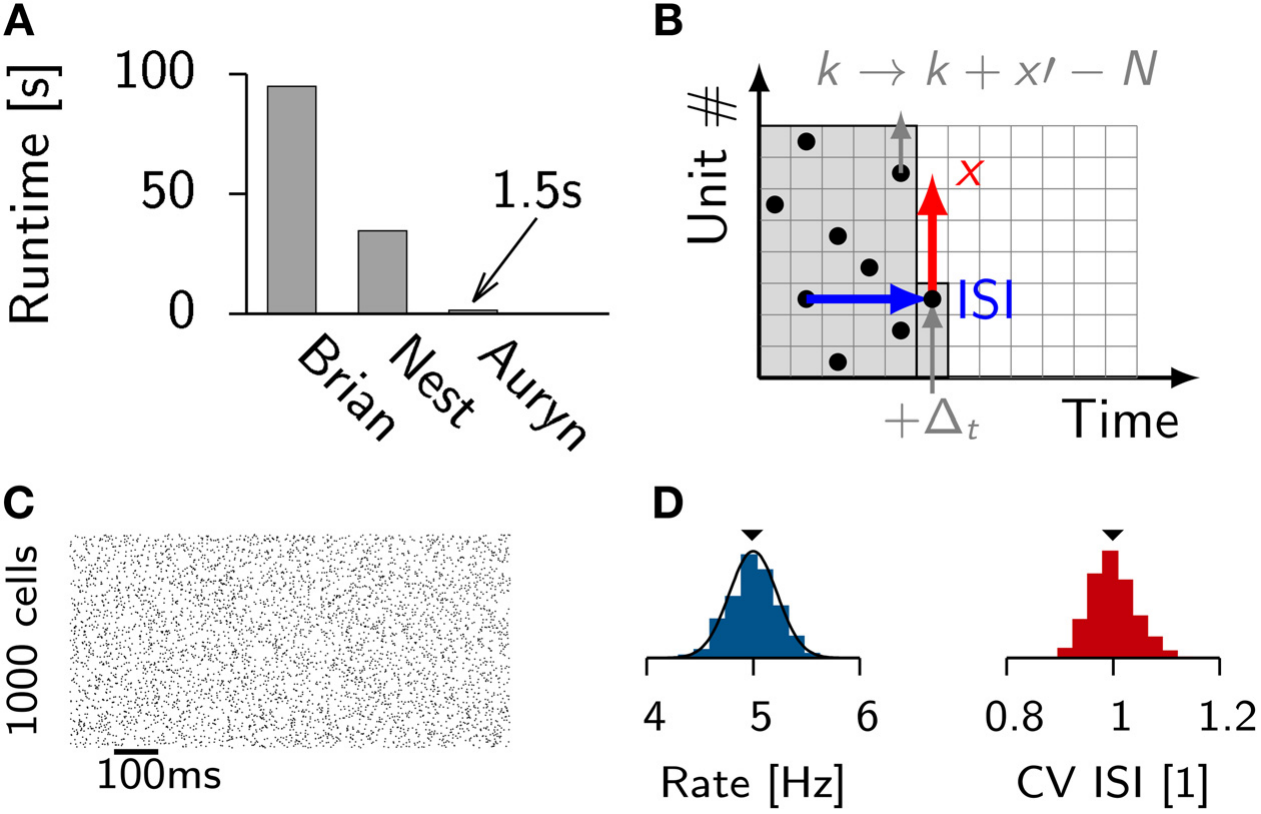
**Efficient generation of Poisson spike trains from a population of input units**. **(A)** Run times of different simulators to generate 100 s long spike trains from 1000 Poisson neurons spiking at 5 Hz. **(B)** Illustration of the algorithm. One typical ISI interval for single Poisson neuron (blue). Typical example of a random stride of magnitude *x* (red arrow) or a stride *x*′ that would lead beyond *N* and is consequently continued in the next time step (gray). **(C)** Spike raster from simulated Poisson spike trains from **(A)**. **(D)** From the same simulation: Distribution of firing rates (left) with the theoretical expectation from the Poisson distribution (solid line). Right: Distribution of coefficient of variation of the ISI (CV ISI). The mean values of the distributions are indicated by arrow heads.

Frequently used functionality in Auryn is implemented in highly specialized classes. One example is the generation of Poisson spike trains. In our simulations, we often use input from a homogeneous population of Poisson neurons which spike at low and identical firing rates. Hence, Auryn comes with an optimized solution for this particular scenario. Most existing simulators come with at least one way to generate Poisson spikes. NEST uses a poisson_generator in combination with “parrot neurons” and the Brian simulator uses PoissonGroup objects. Both examples are more versatile than Auryn's solution. Brian handles different firing rates for each neuron as well as temporal modulation of firing rates. NEST has the ability to recreate the same pseudo random Poisson spike trains independently of the number of cores used. The highly specific implementation in Auryn does not have this flexibility, but it allows for a fast implementation (Figure 1A).

To efficiently generate Poisson spike trains from such a configuration we consider a grid spanned by *N* rows corresponding to the Poisson neurons and discrete-time with bins of size Δ*t* on the *x*-axis (Figure 1B). To create Poisson spikes we could now fill each row of the grid with spikes by drawing exponentially distributed inter-spike-intervals. This can be done on-line, but requires a certain degree of book-keeping because the algorithm has to remember the *N* last spike times of all Poisson units. It is more efficient to fill each column at the very time step when the spikes are needed. This can be done efficiently since the distribution of inter-spike-intervals (ISIs) is the same in x and y-direction. Therefore, all spikes can be generated online when they are needed during the simulation (Figures 1B–D). When a jump leads beyond *N* it is simply continued in the next time step. This way every random number yields a spike.

### 2.5. Implementation of spike-timing-dependent plasticity

Auryn was developed to provide an efficient environment for simulating plastic synapses in recurrent neural networks. To that end we are particularly interested in simulating spike-timing-dependent plasticity (STDP) as it is a form of plasticity commonly found in the brain (Markram et al., 1997; Bi and Poo, 1998, 2001; Zhang et al., 1998; Markram et al., 2011). STDP can be implemented efficiently in an event-based way where synaptic weights only change when pre- or postsynaptic spikes occur (Gerstner and Kistler, 2002b; Morrison et al., 2008).

A broad family of spike-timing-dependent plasticity (STDP) models can be written in the following form (Gerstner and Kistler, 2002b)

(4) $$\begin{array}{l} \frac{dw_{ij}}{dt}=a^{\text{pre}}S_{j}(t)+a^{\text{post}}S_{i}(t)\\ \;+S_{j}(t)\int_{0}^{\infty }W(s)S_{i}(t-s)ds\\ \;+S_{i}(t)\int_{0}^{\infty }W(-s)S_{j}(t-s)ds \end{array}$$

where *a*^pre^ and *a*^post^ are constants, *W*(*t*) is a real valued function with finite support and *S*_*j*_(*t*) is the presynaptic (*S*_*i*_(*t*) the postsynaptic) spike train given as a sum of delta functions *S*_*x*_(*t*) = ∑_*k*_δ(*t* − *t*^*k*^_*x*_) where *t*^*k*^_*x*_ runs over all spike times *k* of neuron *x*. The parameters *a*^pre^, *a*^post^ as well as the window *W*(s) may depend on the momentary value *w*_*ij*_ of the synaptic weights (van Rossum and Turrigiano, 2001; Gütig et al., 2003). Expression (4) describes a piecewise constant function of time with jumps whenever pre- or postsynaptic spikes occur. Note that STDP can also contain higher order terms (Pfister and Gerstner, 2006) which does not influence the key points of our argument. In many situations the window function *W*(*t*) can be well approximated by one or multiple exponential functions. As an example

(5) $$W(t)=\{\begin{array}{ll} A^{+}\exp\left(-\frac{t}{\tau_{A}}\right) & t>0\\ B^{-}\exp\left(+\frac{t}{\tau_{B}}\right) & t\leq 0 \end{array}$$

where *A*, *B*, τ_*A*_, and τ_*B*_ are constants, yields a plausible STDP curve (Zhang et al., 1998; Song et al., 2000; Gerstner and Kistler, 2002a). Whenever the window function can be broken down to exponential shapes, this allows for an efficient on-line implementation by using synaptic traces (Gerstner and Kistler, 2002b; Morrison et al., 2008). A synaptic trace *z*_*i*_(*t*) is a low pass filtered version of the spike train *S*_*i*_(*t*) of neuron *i*. It is described by the linear differential equation

(6) $$\frac{dz_{i}^{x}}{dt}=-\frac{z_{i}^{x}}{\tau_{x}}+S_{i}(t)$$

with associated respective timescale τ_*x*_. In the absence of spikes the solution is a simple exponential decay. Equation (6) can either be integrated time-continuously by multiplication with the constant exp $\left(-\frac{\Delta t}{\tau_{x}}\right)$ in every simulation time step Δ*t* or by using the fact that the analytical solution is known for arbitrary time intervals (event-based update).

By combining Equations (4) and (6) synaptic weight updates can be written as follows

(7) $$\frac{dw_{ij}}{dt}\propto A^{+}z_{j}^{\text{+}}(t)S_{i}(t)-A^{-}z_{i}^{-}(t)S_{j}(t)+a^{\text{pre}}S_{j}(t)+a^{\text{post}}S_{i}(t)$$

which is ideally suited for event-based integration because weight changes only occur at pre- or postsynaptic spike times. To add plasticity to a network simulation, one therefore simply adds the required number of traces [cf. Equation (6)] and the event-based weight update. The simplest implementation of STDP now proceeds as follows: at time *t*_*j*_ of a presynaptic spike of neuron *j* the trace *z*^−^_*i*_(*t*) is read out and the necessary weight update is applied to the weight *w*_*ij*_. Since the postsynaptic trace of neuron *i* can be integrated alongside with the neuronal state no particular care has to be taken for parallel processing.

This changes in two ways in the case of a postsynaptic spike. First, in case of parallel processing the neuron from which the spike originated might not be integrated on the same physical computer. Hence there is no simple way of providing the value of its synaptic trace. Second, the simulator might not offer efficient means of finding all presynaptic partners of a postsynaptic neuron. Doing this efficiently generally costs memory, because each neuron needs to keep a list of all its presynaptic partners.

Per default Auryn takes the simplest approach where at each postsynaptic firing time all associated weight updates are carried out immediately. As a direct consequence, synaptic delays are implemented as purely axonal rather than dendritic delays (i.e., when two neurons spike in the same time step, the postsynaptic spike arrives at the synapse before the presynaptic spike). To be able to provide the value of the trace of any presynaptic neuron at the time of the update Auryn computes presynaptic traces on all nodes in a time continuous way. That means that presynaptic traces are evolved in every time step irrespectively if the value is needed or not. Since every process needs to keep track of all presynaptic traces, it also means that some redundant work is being done.

Auryn alternatively supports an event-based approach. This approach exploits the fact that in the absence of spikes, the solution to Equation (6) is an exponential decay. Since the event-based trace update cannot be vectorized efficiently this only provides an advantage at low firing rates. Since, the increased overhead due to the use of the exponential function generally seems to outweigh the advantages of this approach, which is why Auryn chooses by default time-continuous updates of presynaptic traces.

In contrast to that NEST uses an event-based approach in which synaptic weight updates are only carried out at the arrival times of presynaptic spikes (Morrison et al., 2007). To do this, each neuron stores its past firing history in a small buffer. Whenever a presynaptic spike occurs, all post-pre updates are applied retrospectively in a batch. Since for each update all quantities appearing in Equation (7) have to be known, the retrospective update requires to keep track of these values or to compute them on-the-fly when needed. Because all recent postsynaptic firing times are available at the time of arrival of a presynaptic spike, this approach offers more flexibility with respect to whether spike transmission delays are interpreted as axonal or dendritic delays. On the downside, the storage of postsynaptic spike times as well as the evaluation of the exponential function required for the on-the-fly computation of synaptic trace values causes overhead.

## 3. Results

**Figure 2:**
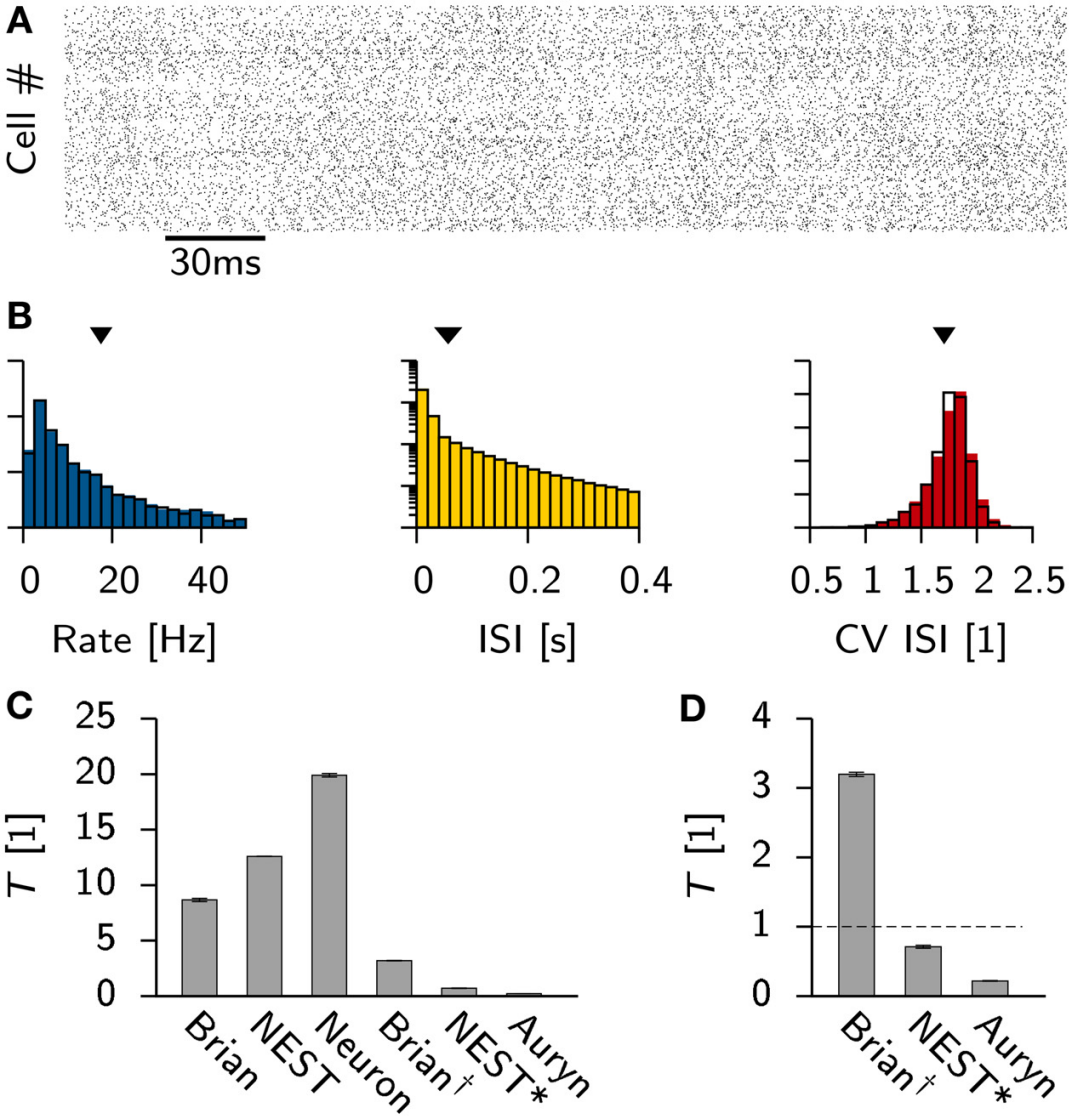
**Vogels-Abbott benchmark**. **(A)** Spike raster of the excitatory cells in the network as simulated with Auryn. **(B)** Distribution of the firing rates (left), the inter-spike-intervals (middle) and the the coefficient of variation (right). Solid color bars are the results obtained from Auryn. The black lines is the output from NEST. Mean values of the distributions are indicated by arrow heads. **(C)** Relative run times *T* of different simulators and different configurations for simulating 60 s of the Vogels-Abbott benchmark. The values for Brian, NEST and Neuron are given for the respective simulators in their standard configuration. Brian^†^ corresponds to Brian with performance optimizations enabled. NEST^*^ is the same as NEST, but using a minimal forward Euler solver. **(D)** Zoom on the comparison of Brian^†^, NEST and Auryn. The dashed line indicates a real-time simulation.

To compare the possible simulation speed of balanced network models using different publicly available simulators and standard hardware (Methods) we adapted the conductance based Vogels-Abbott network (Vogels and Abbott, 2005), which has been used as a benchmark in the past (Brette et al., 2007; Sharp and Furber, 2013). In particular we implemented the same network in Neuron (Carnevale and Hines, 2006), NEST (Gewaltig and Diesmann, 2007), Brian (Goodman and Brette, 2008) and our own simulator Auryn (Methods). The network was tuned initially to a parameter regime in which the model exhibits stable asynchronous irregular activity over extended periods of time (Figure 2A). We ran network simulations for each simulator in their standard configuration and ensured that the different simulations produced comparable results (Figure 2B). All simulations were run for 1 min of simulated time, using only a single core. Only the execution time of the main simulation procedure was timed, while time consumed to set up the network, or for writing data to disk, was ignored.

The observed individual run times differ substantially between simulators (Figure 2C), which can have multiple different reasons. First, in the standard configuration of a simulator certain performance options might be turned off for compatibility reasons. In the case of Brian for instance, enabling such features resulted in a significant performance boost, which led to a speed-up of the simulation by more than a factor of two (Brian^†^, Figures 2C,D). Second, different simulators use different numerical solvers for the differential equations which describe the neural dynamics. For instance, in the present simulation NEST uses a Runge-Kutta-Fehlberg 4(5) solver (Fehlberg, 1970), whereas Brian, as well as Auryn, rely on the simpler forward Euler method. While the former yields higher precision, it also is computationally more expensive.

To characterize the difference between the two methods, we reimplemented the neuron model in NEST using forward Euler integration (we will refer to this implementation as NEST^*^ for disambiguation). Consequently the same simulation was repeated, which resulted in more than a ten-fold decrease in simulation time (Figures 2C,D).

Since this speed-up comes at the cost of precision it is natural to ask what precision is required. There is no general answer to this question. All numerical integrators only approximate the dynamics of the true dynamical system. Since in most balanced networks small deviations of the initial values grow exponentially over time (van Vreeswijk and Sompolinsky, 1996) it is virtually impossible to simulate the same temporal evolution twice using different integration methods. Nevertheless, all simulations approximate the same network at the macroscopic level which can be characterized by meaningful network statistics. The latter can be compared across simulators (Davison et al., 2009; Henker et al., 2012).

To investigate the impact of precision on the network statistics of the Vogels-Abbott benchmark we used the simulation data obtained with NEST as a reference model and compared the network statistics (Figure 2B) with the results obtained with Brian and NEST using a two sample Kolmogorov-Smirnov (K-S) test. In particular we found that none of the sampled rate distributions were significantly different (*p* > 0.5 in all cases). However, the K-S test indicated statistically significant differences for the coefficient of variation of the ISI distribution (CV ISI) between Auryn and the other simulators (*p* < 0.003) whereas differences between Brian and NEST were not significant (*p* > 0.2). A similar comparison of the ISI distributions showed that all samples from all simulators differed significantly (*p* < 2 × 10^−60^ in all cases).

To verify that all requirements for the K-S test with respect to independence of the samples and stationarity were met, we computed the K-S statistic and corresponding *p*-values for two samples of the rate and the CV ISI distribution from the same simulation (20–30 s and 40–50 s). Both did not show significant differences (NEST: *p* > 0.12; Brian: *p* > 0.45). Similar to earlier findings (Davison et al., 2009), the same analysis on two independent samples of the ISI distribution showed, that the samples were significantly different (NEST : *p* < 1.3^−85^; Brian: *p* < 6.6^−8^).

**Figure 3:**
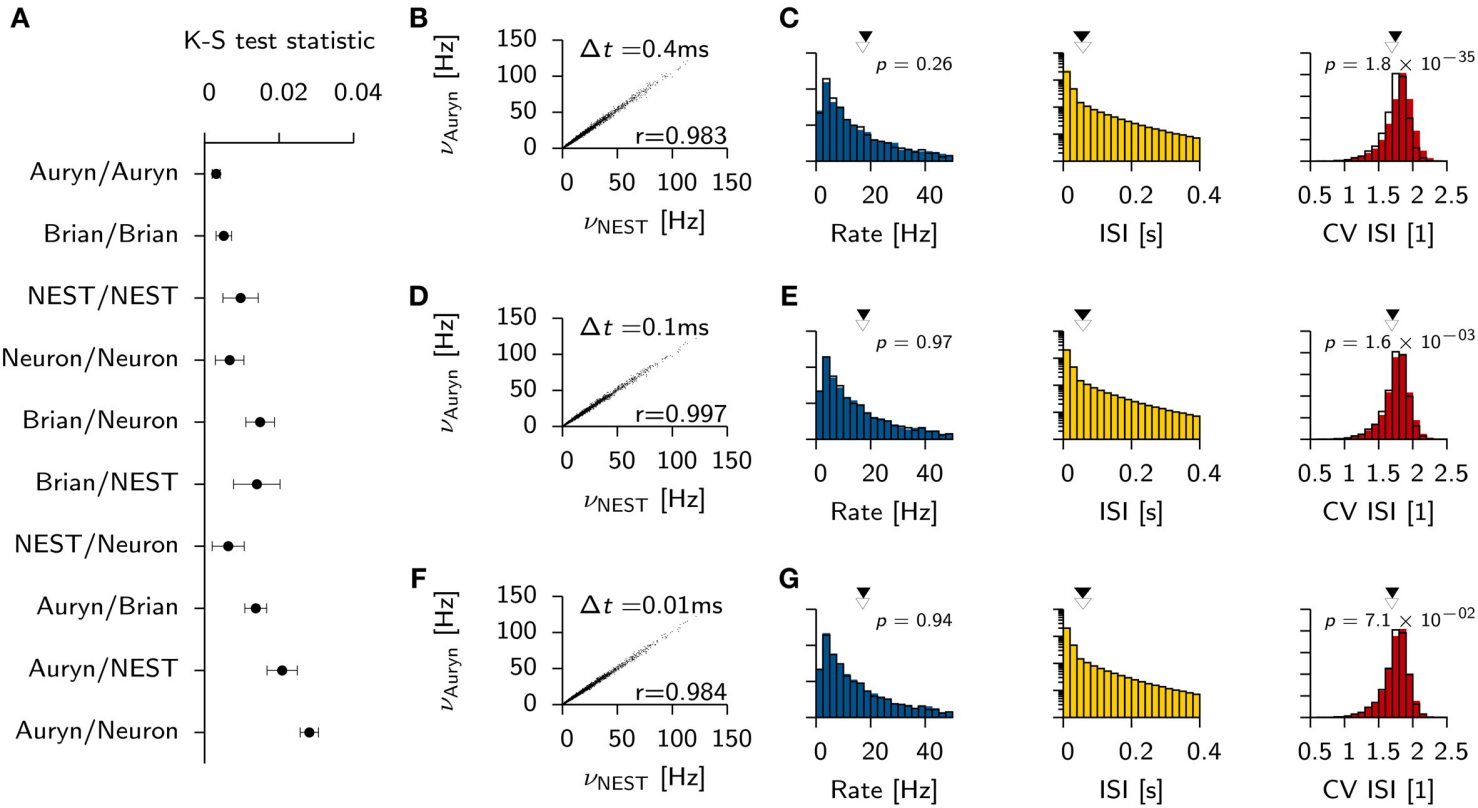
**Effect of simulator and integration step size on network statistics**. **(A)** Mean K-S test statistic *D* comparing various combinations of ISI distributions obtained from the Vogels-Abbott benchmark with different simulators (integration time step Δ*t* = 0.1 ms). Each data point corresponds to the mean value of *D* computed from 10 pairs of independent samples of the ISI distribution. Error bars signify the standard deviation. **(B)** Individual neuronal firing rates from Auryn simulation with Euler step size Δ*t* = 0.4 ms against reference data set obtained with NEST [Runge-Kutta-Fehlberg 4(5), 0.1 ms time step]. *r* is Pearson's correlation coefficient of the two data sets. **(C)** Histograms of network statistics: Rate (left), inter-spike-interval (ISI, middle) and the coefficient of variation of the ISI (CV ISI, right) for the simulation with Δ*t* = 0.4 ms. Solid color bars: results from Auryn simulation. Mean value indicated by solid arrow head. Black lines: results from NEST simulation. Mean value given by empty arrow head. *p*-values from two sample Kolmogorov-Smirnov test on the raw data before binning. **(D,E)** Same as top row **(A,B)** but for Δ*t* = 0.1 ms. **(F,G)** same as before, but for Δ*t* = 0.01 ms.

To gain quantitative insight into the variability and the differences of the ISI distribution for a single simulator and across different simulators we computed the mean K-S statistic *D* for 10 pairs of independent samples from the ISI distributions (Figure 3A). Consistent with earlier findings (Davison et al., 2009) the comparison between NEST and Neuron yields *D* values that are comparable to the ones seen from independent samples from within the same simulation performed with either simulator. Auryn and Brian, which use less precise integration schemes, exhibit larger *D* values in direct comparison to Neuron or NEST or to each other, but comparably low mean values and fluctuations when compared to themselves individually (Figure 3A).

To check whether or not the differences between simulators using different integration schemes could be made negligible, we ran additional simulations for three different Euler time steps in Auryn (Δ*t* = [0.4, 0.1, 0.01] ms; reference simulation: NEST with Δ*t* = 0.1 ms). We then compared the firing rates of the excitatory neurons in the simulation with Auryn to the ones observed in the reference simulation and find that they are highly correlated regardless of the time step used (Figures 3B,D,F; Supplementary Figure S1). Moreover, the observed distributions of the inter-spike-intervals (ISI) and the CV ISI are qualitatively similar in all cases (Figures 3C,E,G; Supplementary Figure S1). Detailed comparison (K-S test) shows that the observed samples of the rate distributions do not differ significantly from the reference data (Figures 3B,D,F). However, the observed samples of the CV ISI distributions are different for the 0.4 and the 0.1 ms time step case. In the case of the 0.01 ms time step the difference is not significant (*p* > 0.07; Figure 3F). Direct comparison of the K-S statistic on the ISI distributions resulted in larger *D* values than the ones obtained earlier (Figure 3A) due to the change of the temporal grid that spikes are aligned to (not shown).

Our analysis illustrates that differences in the integration algorithm have a measurable effect on macroscopic network quantities when time steps well below 1 ms are used. To speed up simulations further, an increase of the integration time step should therefore be avoided. Otherwise, a too large integration time step will result in synchronization effects or other artifacts which can be mistaken for real network effects (Hansel et al., 1998). Throughout the remaining manuscript we set the time step to 0.1 ms, which is chosen as a compromise between speed and accuracy. In conjunction with the particular network and the hardware used, this allowed us to simulate the Vogels-Abbott benchmark faster than real-time when using NEST^*^ or Auryn, while the simulation in Brian^†^ was approximately three times slower than real-time.

These data show that when precision is not a primary concern, real-time simulations of small neural networks are well in reach using standard hardware and simulation tools. While real-time simulations are important in situations were the network is to interact with the real word—as for instance in robotics applications or in certain *in-vitro* experiments, for plasticity studies it is often desirable to speed-up simulations as much as possible. To achieve even higher simulation speeds most simulators used in this study (with the exception of Brian) can run simulations in parallel.

### 3.1. Parallel simulations

**Figure 4:**
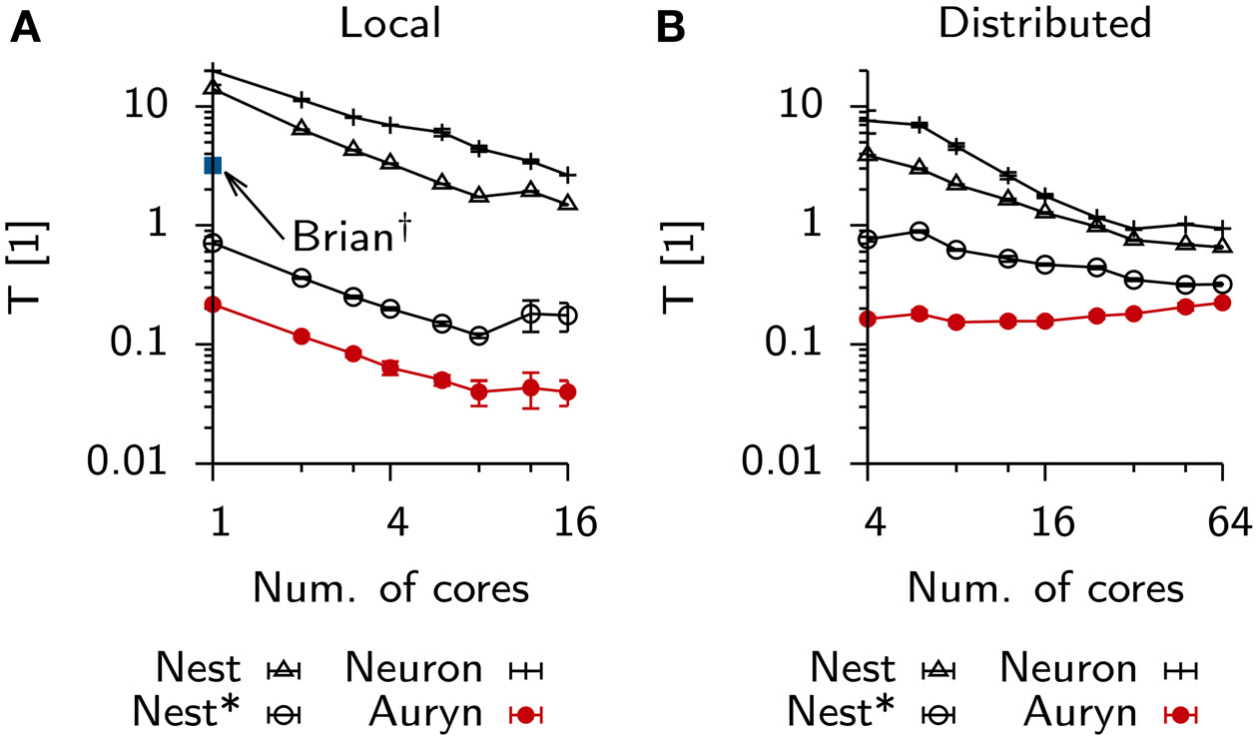
**Scaling behavior Vogels-Abbott benchmark**. **(A)** Relative run times of simulators allowing parallel execution for 60 s of benchmark simulation as a function of the number of cores used in a single machine with 16 physical cores (local). **(B)** Same as **(A)**, when simulations are run distributed on four nodes connected via Ethernet.

To analyze how much further the present example of the Vogels-Abbott benchmark could be sped-up by using parallelism, we repeated the above measurement using multiple cores locally on a single machine (Figure 4A) or distributed using four identical machines connected via Ethernet (Figure 4B). In the case of local execution it was possible to increase the execution speed by about one order of magnitude. In particular we observed strong scaling which stopped when 12 out of the 16 physical cores on a single machine were used. In the distributed case all simulators, except Auryn, exhibited decreased execution times, while parallel execution in Auryn even slowed down the simulation. However, despite the fact that Auryn's execution times increased monotonously with increasing numbers of cores, it was still systematically faster than the other simulators. Compared to the local case, the overall scaling behavior in the distributed case was less uniform across simulators and number of cores used (note the different and changing slopes in the log-log plot; Figure 4B).

**Figure 5:**
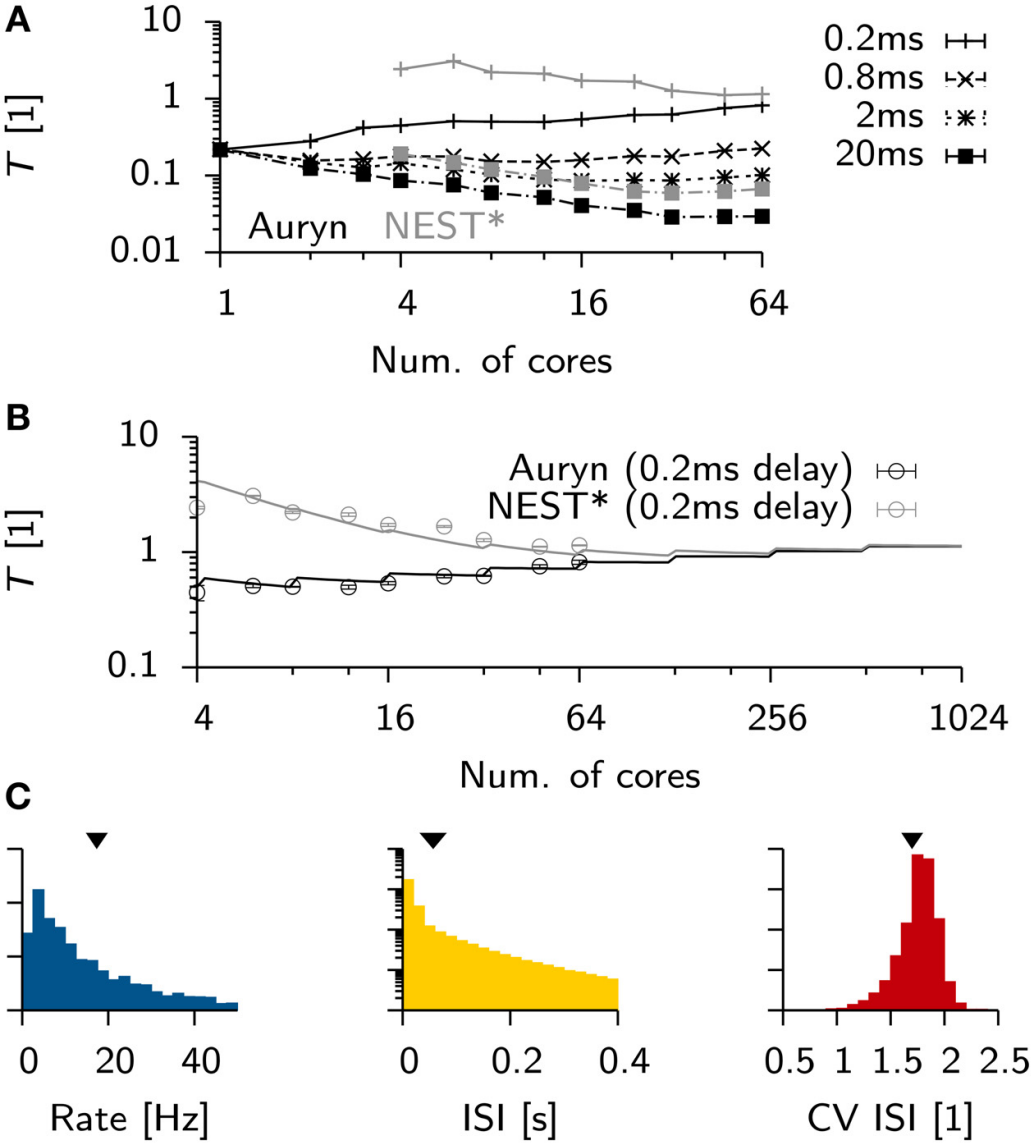
**Scaling of Vogels-Abbott benchmark for different synaptic delays**. **(A)** Relative run time *T* of Vogels-Abbott benchmark simulated with Auryn for different synaptic delay values (black). Gray: The two most extreme measurements 0.2 and 20 ms are shown for NEST^*^ for reference. Only data points for 4 cores and more are plotted for clarity. **(B)** Data points for the 0.2 ms delay case in **(A)** with simple run time model extrapolated to hypothetical numbers of cores [solid lines; cf. Equations (2) and (3)]. The full model was first fitted to the Auryn data points. The gray curve was obtained from this model by only adjusting the parameter α in the model. **(C)** Network statistics for network with 20 ms synaptic delay. From left to right: Rate distribution, inter-spike-interval distribution (ISI) and coefficient of variation of the ISI (CV ISI). Arrowheads at the top indicate mean values of the respective distribution.

To see how these results generalized for different settings of the minimum synaptic delay in the Vogels-Abbott benchmark, we ran additional simulations in Auryn and NEST^*^ for synaptic delays ranging from 0.2 ms up to 20 ms. We found that an increase in synaptic delays improves scaling on larger numbers of cores (Figure 5A). Similar to the above findings, shorter synaptic delays resulted in monotonously increasing run times in Auryn, whereas at this point the run times in NEST^*^ were still decreasing. It is not clear whether run times of NEST^*^ or Auryn could cross over for larger numbers of cores or if they reach a common asymptote. The latter case can be captured in simple run time model which assumes comparable communication delays for both simulators (Figure 5B; Methods). While the plausibility of synaptic delays in the order of 20 ms is questionable, the Vogels-Abbott benchmark, regardless of this, generated network activity which was in qualitative agreement with a network with a 0.8 ms synaptic delay (Figure 5C).

**Figure 6:**
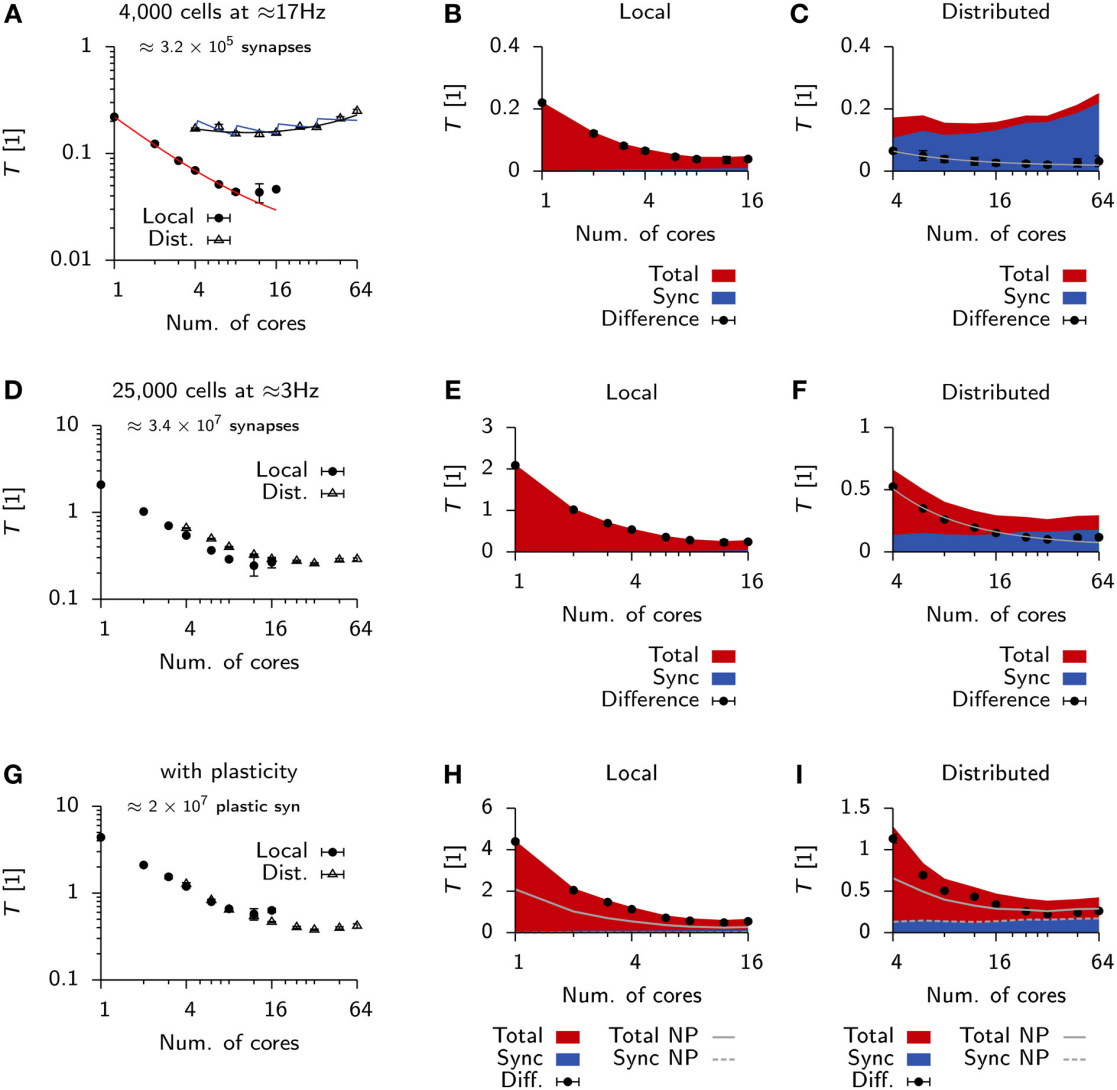
**Scaling behavior in Auryn**. **(A)** Scaling of relative run time in the Vogels-Abbott benchmark on a single machine and when run distributed over four machines. Solid red line: RMS fit of Expression (2) to local run time data. Blue and black lines: RMS fit of Expression (2) and (3) respectively to distributed run time data. Error bars represent the standard deviation of the data. **(B)** For the same simulation as in **(A)**. Time spent on total computation (red) and the fraction spent on synchronization (blue) for different numbers of cores used locally. Black bullets: Difference between the two. **(C)** Same as **(B)**, but when run distributed over four machines. Gray line: RMS fit of $f(x)=\frac{A}{x}+B$ to difference data points. **(D)** Scaling of relative run time in the 25 k cell network without plastic synapses on a single machine and when run distributed over four machines. **(E,F)** Same as **(B,C)** but for the 25 k cell network from **(D)**. (**G–I)** Same as before, but for the 25 k cell network with plastic excitatory-to-excitatory synapses. **(H,I)** Solid gray line: Total from **(E,F)** for reference. Dashed line: Sync from **(E,F)** for reference.

Qualitative differences in scaling behavior for the Vogels-Abbott benchmark can be understood using a simple run time model (Methods) which takes into account communication delays. The same model also captures the observed differences between local an distributed simulations (Figure 6A). To further investigate the effect of communication delays and to study whether they pose indeed the limiting factor for distributed simulations, we inserted additional profiling code into the Auryn simulation which allowed us to directly measure the time spent on communication, i.e., synchronizing the different processes (sync). This measurement confirmed that communication time was negligible in the local case (Figure 6B), whereas it became the dominating contribution to total run time for the distributed simulation (Figure 6C). While the increase in overall run time can mostly be attributed to the sync time, we found that also the difference between total run time and sync time does not scale as $\sim \frac{1}{n}$ where *n* is the number of cores, but shows saturating behavior (Figure 6C).

We were wondering how this behavior would change for larger and more memory intensive simulations. To that end we simulated a 25,000 cell network with a mean population firing rate of 3 Hz (Methods). In line with the expected higher computational cost, due to the larger number of neurons, we measured longer run times on a single core (Figure 6D) than for the Vogels-Abbott benchmark.

Similarly to the Vogels-Abbott benchmark, strong scaling ends for distributed runs (Figure 6D) and run times cannot be decreased below *T* ≈ 0.2 (Figures 6D,E). However, in the distributed case the total run time is no longer dominated by sync time, but a significant fraction of the total time is spent on computation (Figure 6F).

To test how the same simulation scales with synaptic plasticity we added triplet STDP (Pfister and Gerstner, 2006) to all excitatory-to-excitatory synapses (Methods; Zenke et al., 2013). In this case the differences in scaling behavior between the local and the distributed simulation become negligible (Figure 6G). However, we measured a small increase in sync time for the local plastic simulation (Figure 6H), while the sync time was virtually identical for distributed runs of the plastic and the non-plastic network (Figure 6I).

It has been described previously in plastic spiking network simulations, that a substantial part of communication time is spent at the implicit barrier in the AllGather directive, which is commonly used to communicate spikes between nodes, and not the actual communication latency (Ananthanarayanan et al., 2009). In particular that means that processes which finish their computation earlier have to wait for slower processes at every sync instance. We were wondering to what degree this effect was present and measurable in our simulations.

**Figure 7:**
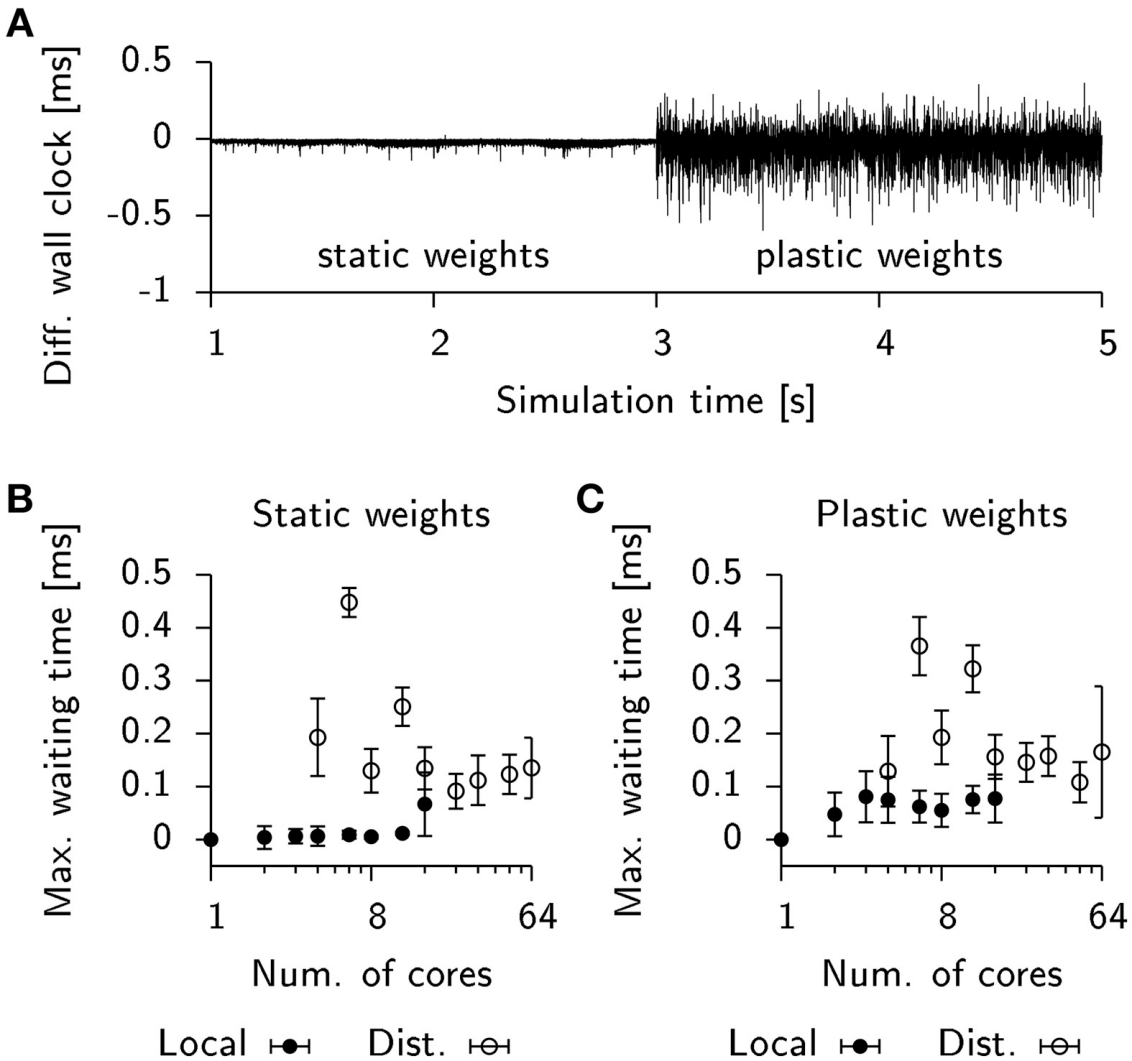
**STDP affects load-balancing negatively**. **(A)** Time difference of wall clock times between a total of two processes from the 25 k cell network simulation when run locally on a single machine. Initially, weight updates are not computed (static weights). Plastic weight updates are switched on at *t* = 3 s. **(B)** Maximum wall time difference between two processes before synchronization for the static network. Filled bullets: Data from local execution. Empty bullets: Data from distributed runs. **(C)** Same as **(B)**, but for the plastic network. Error bars show the standard deviation.

To address this question we inserted additional code into the Auryn simulations of the 25 k cell network to record the wall clock time of every process after each integration time step. Figure 7A shows the difference in wall time between two processes in the same simulation in which plasticity was initially disabled. The observed time differences are small, which suggests that the processes roughly have the same execution time per duty cycle (the simulation performs an AllGather every 0.8 ms of simulated time). This, however, changes once plastic updates are enabled (Figure 7A). The number of spikes occurring in each process per duty cycle varies stochastically which in turn influences the execution time of the code responsible for plasticity updates. Since the number of spikes in different processes can vary independently, this gives rise to large fluctuations of the difference between the wall times of both processes.

To estimate the impact of this effect on run times in our simulation we extracted the wall timings before each AllGather and determined their respective temporal offsets. We then computed the maximum temporal offset before each sync which correlates with the waiting time until the last process arrives at the barrier. For simulations of the static network these timings were close to zero in the case of a local simulation or fluctuating at about 0.1 ms for a distributed simulation (Figure 7B). When plasticity was enabled, the waiting time measurably increased to about 0.05 ms in the local simulations. However, the effect was difficult to observe in the case of a distributed simulation due to the overall high fluctuations in waiting time (Figure 7C). This suggests that for distributed simulations the implicit barrier does not represent the crucial bottle neck on standard hardware, but rather actual communication delays constitute the limiting factor.

So far we have isolated the sync time and found it to be a significant contributor to run time especially in distributed simulations. To identify other potential sources which end strong scaling in our simulations we profiled the above simulations using gprof (Graham et al., 1982). Specifically we recorded run time information for the Vogels-Abbott benchmark and the 25 k cell network (static and plastic). We then hand-labeled the most costly functions according to three categories: First, all functions involved in integrating the neural state variables and synaptic traces: “Evolve.” Second, explicitly excluding the sync time, all functions contributing to spike propagation: “Propagate.” Third, the specific contribution of spike propagation and weight update at plastic synapses “Triplet.”

**Figure 8:**
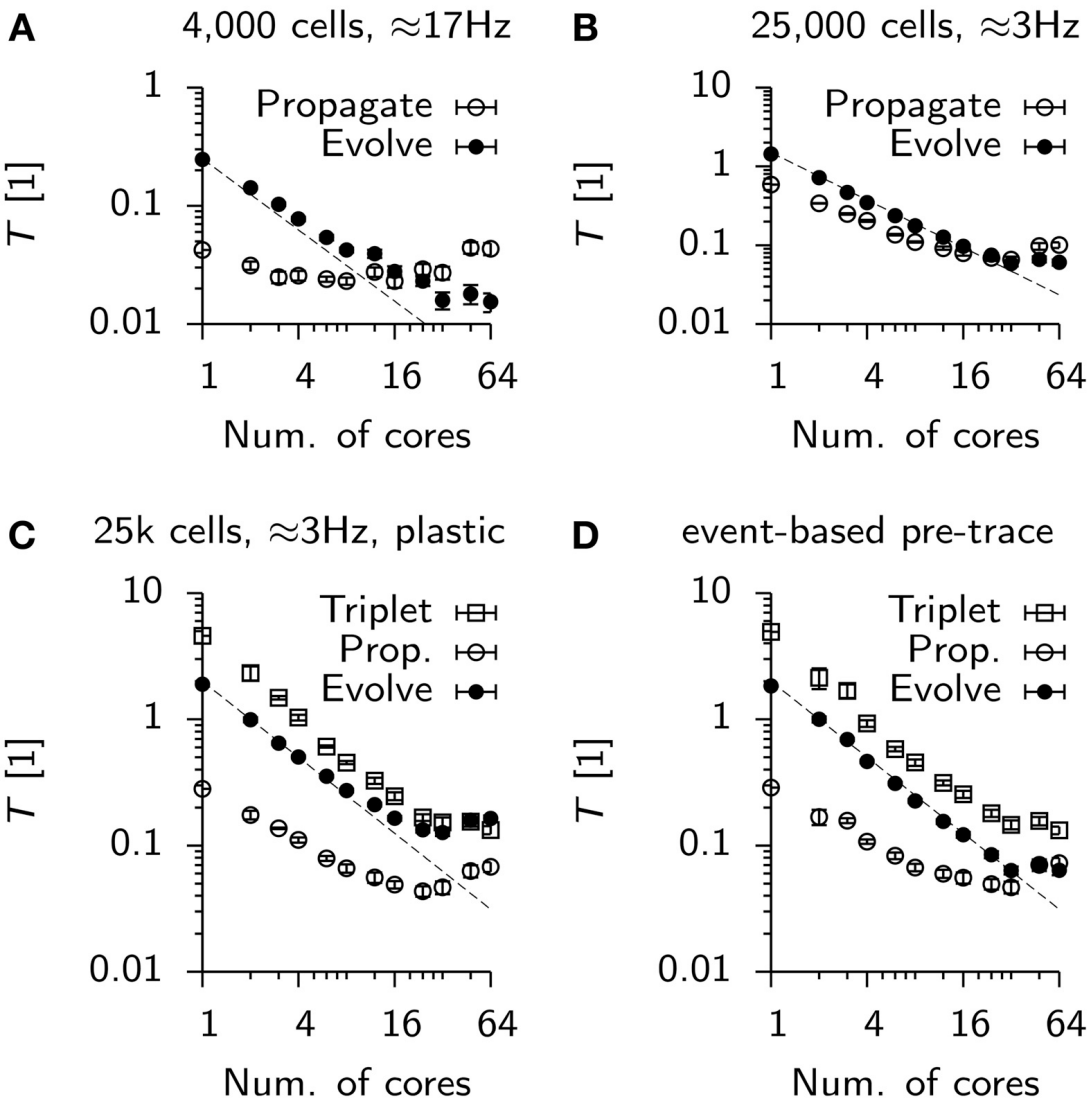
**Scaling of parallel simulations without communication times**. **(A)** Time spent by each parallel process in a designated class of functions. Propagate: Functions involved in delivering spikes to postsynaptic neurons (not including synchronization). Evolve: Functions serving directly or indirectly the integration of continuous neuronal or synaptic state variables. Data points represent mean values averaged over all processes of a simulation. Error bars give the standard deviation from this average. The dashed line is given for reference. It represents perfect strong scaling. **(B)** Same as **(A)**, but for the static 25 k cell network. **(C)** Same as **(B)**, but for the plastic network. The new function class “Triplet” includes functions involved in computing weight updates and the propagation of excitatory-to-excitatory spikes. Note, that the evolution of synaptic traces is counted in “Propagate.” **(D)** Same as **(C)**, but with event-based update of the presynaptic trace variables.

For the Vogels-Abbott benchmark we find that Evolve scales slightly sub-linearly (Figure 8A), while the contribution of Propagate is approximately constant and even rises for high numbers of parallel processes. Notably, while Evolve takes the lion's share of computation time for low numbers of cores, the behavior changes at around 16 cores, where the run time is dominated by Propagate (Figure 8A).

In the case of the 25 k cell network, initially Propagate and Evolve scale close to linearly until strong scaling breaks for about 48 to 64 cores (Figure 8B). The situation is worse in the plastic network in which both, Triplet and Evolve saturate at about *T* ≈ 0.15, whereas Propagate even increases from 24 cores onwards (Figure 8C). The deviation of Propagate from strong scaling is most likely linked to the fact that in its default configuration, each process in the simulation maintains and integrates a copy of all presynaptic traces to implement triplet STDP (note that synaptic traces are for technical reasons counted against Evolve). When the presynaptic trace is switched to event-based integration, strong scaling is restored up to 32 cores for Evolve (Figure 8D). The associated cost increase in Triplet, however, makes the overall simulation slower, which is why event-based integration is disabled per default in Auryn.

### 3.2. Computational cost of alternative STDP implementations

While the fastest run times with Auryn were achieved with an STDP implementation which purposefully breaks strong scaling, NEST uses a different approach to STDP. In particular, in its standard implementation synaptic weights are updated only at the arrival of a presynaptic spike. All previous weight updates caused by postsynaptic spikes are then executed in a batch for which presynaptic traces are computed retrospectively on the fly (Methods). In this approach scaling is preserved because no copies of presynaptic traces have to be kept locally, but the computation of synaptic updates becomes more expensive.

To study which approach leads to faster run times we compared the performance of plastic network simulations in Auryn and NEST. To do so without having to implement new plasticity rules in NEST, we limited our study to code that already existed. In particular we adapted two example simulations that come with the current NEST release (Gewaltig and Diesmann, 2007; Gewaltig et al., 2012). Both simulations implement the same balanced random network model based on work by Brunel (2000). In one case all connections are static whereas in the other case excitatory-to-excitatory connections evolve according to a weight dependent STDP rule.

**Figure 9:**
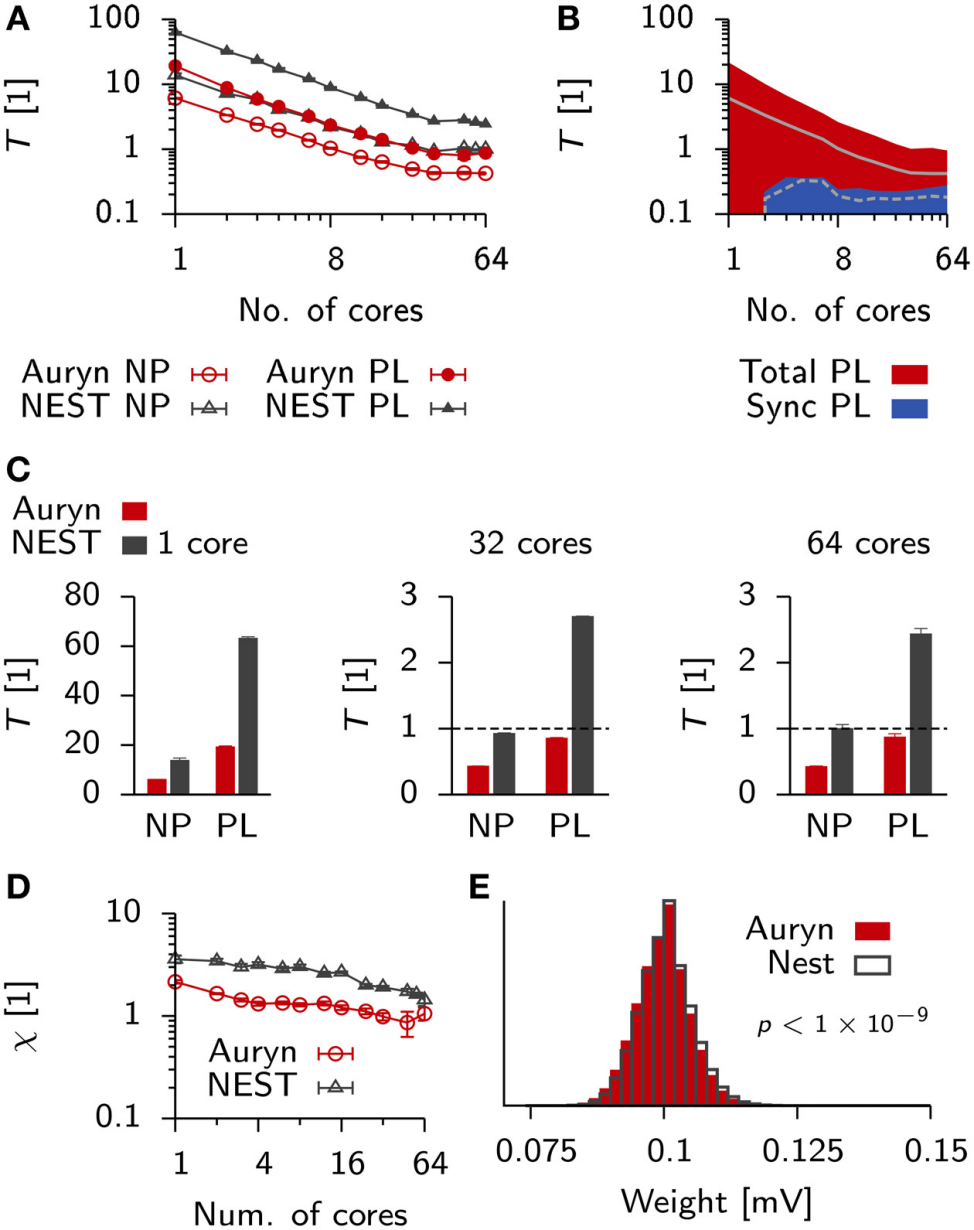
**Comparison of run times in balanced networks with plasticity**. **(A)** Scaling behavior of the relative run time *T* for a classical balanced network model (Brunel, 2000) when simulated in NEST (black) and Auryn (red). The measurement was done for the non-plastic (NP) network and a network with multiplicative STDP (PL). **(B)** Total run time (red) and time used for synchronization (blue) for the plastic simulation using Auryn. **(C)** Bar plot of the end point values in **(A)**. Single core (left), 32 cores (middle) and all 64 cores (right). Dashed line: real-time simulation. **(D)** Relative difference between run time of the plastic and non-plastic network for Auryn (red circles) and NEST (gray triangles). **(E)** Final weight distribution after 20 s of simulated time for runs with learning rate λ = 1 × 10^−2^ using Auryn (red) and NEST (gray). *p*-value computed from Kolmogorov-Smirnov test comparing the two samples.

We created the same network model in Auryn. For a better comparison we wrote an Auryn class comparable to the NEST poisson_generator and implemented the same neuron model and integration scheme as used in NEST (both classes are openly available in the Auryn code base). However, due to differences in the implementation of STDP (Methods), Auryn assumes a purely axonal synaptic delay, whereas NEST assumes a dendritic delay. Network simulations were run for 20 s simulated time with low learning rates to avoid that plasticity influences the firing statistics over the time course of the simulation. The resulting network activity of both implementations was comparable at ≈ 43 Hz population firing rate. We ran both simulations distributed over four machines for varying numbers of cores. The respective run times of the plastic (PL) and the non-plastic (NP) configuration were recorded (Figure 9A). On a single core the addition of STDP caused an increase of the run times by a factor of ≈ 2.5 for Auryn (≈ 3.5 for NEST, Figure 9C, left).

Both simulators showed good initial scaling behavior, which saturated quickly (Figures 9A–C). When going from 32 to 64 cores, run times only decreased marginally (Figure 9C). Auryn achieved real-time or faster simulation speed for the NP and PL configurations (Figure 9C). When plotting the relative run time increase caused by plasticity $\chi =\frac{T_{\text{PL}}-T_{\text{NP}}}{T_{\text{NP}}}$ both methods did not exhibit any apparent scaling behavior (Figure 9D). We repeated the above measurement for Auryn using the event-based strategy for presynaptic trace update and did not find any significant differences (data not shown).

Finally, to compare the outcome of an actual plastic simulation we ran simulations in NEST and Auryn with a learning rate of λ = 1 × 10^−2^ and compared the resulting steady state weight distributions (Figure 9E). Similarly to the comparison of network statistics (cf. Figure 3) we found the resulting samples to be qualitatively similar, but significantly different (K-S test: *p* < 10^−9^), which could be due to the contrasting locations of the synaptic delay (axonal or dendritic) in the differing STDP implementations of Auryn and NEST (Methods).

**Figure 10:**
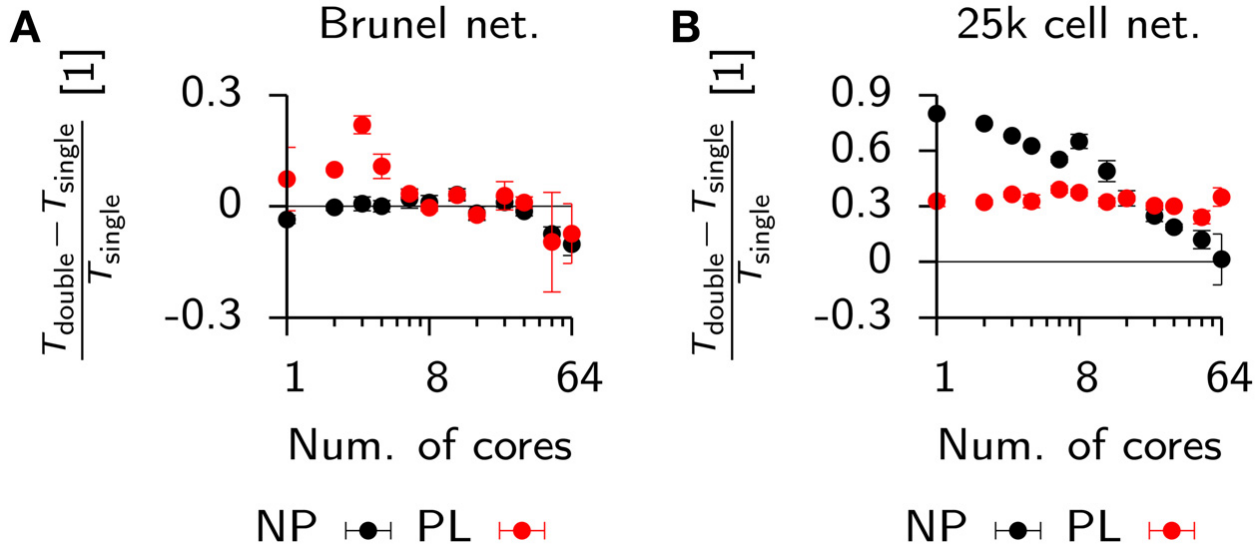
**Effect of floating point precision and the use of SIMD instructions on the run time of network simulations**. **(A)** Relative run time difference between the plastic Brunel benchmark simulation (cf. Figure 9) in Auryn using double vs. single-precision floating point variables. Non-plastic (NP) network (black), plastic implementation (PL; red). **(B)** Same as **(A)**, but for the 25,000 cell network (cf. Figure 6) which uses a fully vectorized neuron model.

Despite the similar implementations (i.e., neuron model and Poisson generator), run times in NEST were about a factor of two (or ≈ 2.5× for PL simulations) longer than the same simulations in Auryn. To check whether the observed run time differences between Auryn and NEST were a consequence of the use of single-precision floating point arithmetic over double-precision, we repeated above run time measurements using double-precision variables in Auryn. The change did not have a strong impact on the steady state weight distribution, which remained significantly different from the reference obtained with NEST (K-S test: *p* < 10^−9^; data not shown). However, the use of double-precision arithmetic caused an increase by about 10–20 percent in run times for low numbers of cores (< 8), these differences became negligible when more cores were used (Figure 10A). Hence, differences in run time between Auryn and NEST, in the present simulation, were not due to differences in floating point precision. However, they were not solely due to differences in the STDP implementations either, because the non-plastic networks alone exhibited different run times (Figure 9C). For both simulators the increase in run time due to plasticity was substantial and it was about three times larger in NEST than in Auryn. This suggests, that for the medium-sized network investigated here, an STDP implementation in which weights are updated at the occurrence of each pre and postsynaptic spike (e.g., Auryn; Methods) can be faster than a paradigm in which weight updates are performed for presynaptic spikes only (e.g., NEST; Methods). It should be noted, however, that the speed-up in the present example comes at the cost of flexibility with respect to how a given STDP implementation can interpret spike transmission delays as axonal or dendritic delays (Methods).

The floating point precision used in a simulation can affect performance in two major ways. First, double-precision variables take up twice the amount of memory compared to single-precision floating point values. This reduces the number of values that fits into a single cache line by a factor of two and thus makes time consuming cache misses more likely. Partly, this limitation can be overcome by using an increased number of parallel processes running on separate physical cores, which increases the total amount of physical cache memory and thus reduces the possibility of cache misses. Second, many modern CPUs offer “single instruction multiple data” (SIMD), which allows to apply the same arithmetic operations on multiple floating point values at once, provided the compiler detects this parallelism and uses SIMD. In the present study we used Intel CPUs equipped with “Streaming SIMD Extensions” (SSE). The SSE instruction achieves its highest performance for single-precision arithmetic. Specifically, SSE registers can either process two double-precision or four single-precision floating point numbers at once (SSEv2 and newer), thus predicting a maximally achievable speed-up for single-precision by a factor of two.

The fact that we only observed run time differences between single and double-precision implementations for small numbers of cores, suggests that these differences are due to limits in the cache architecture, which would explain their disappearance when more than 8 cores are used (Figure 10A). This, however, also suggests that the present code does not make extensive use of vectorized SIMD instructions, because otherwise a significant difference in run time should prevail even when many cores are used.

In the present simulation we used a neuron model which was implemented after the example of NEST for better comparison. In this paradigm each neuron is processed individually (i.e., first synaptic conductances are computed, then the membrane potential is updated and finally the membrane potential is checked for threshold crossings), which prevents it from being compiled into SIMD instructions. In contrast to that, most of Auryn's default models, such as the one deployed in the above 25,000 cell network simulation (cf. Figure 6), use an explicit vectorized formulation in which each atomic arithmetic operation on the neuronal state variables is applied to all neurons before the next operation follows. This vectorized approach allows the compiler to detect and efficiently use SIMD.

To quantify the performance gain due to vectorization, we repeat the time measurements in the plastic 25,000 cell network (cf. Figure 6), which natively compiles using SIMD. The relative comparison shows a substantial increase in run time for double-precision simulations over code using single-precision arithmetic (Figure 10B). These results are consistent with the idea that SIMD operations used for the neuronal state integration are sped up by a factor of two for single-precision arithmetic (maximum achievable difference: 100%).

In summary this analysis shows that SIMD improves performance notably given that the neuronal model code is designed with vectorization in mind. Moreover, for medium size networks, a simple weight update approach to STDP such as used in Auryn is faster than the method NEST uses while yielding similar results. At present it is not clear if or how STDP implementations could be made more efficient to increase simulation speeds even further.

## 4. Discussion

In this paper we have shown that small or medium size recurrent networks with STDP can be simulated in, or faster than real-time if performance-optimized parallel code is used. However, we also show that the margin for speed-up through parallelization on standard hardware is limited due to finite communication delays and the deviation from strong scaling in the mechanisms of spike propagation.

In particular we compared the run times of several standard simulators (Brian, NEST and Neuron) and our own simulator Auryn, when simulating a classic Vogels-Abbott benchmark network. We illustrated that the choice of integration algorithm has a considerable effect on performance. Specifically simulation speed can be increased substantially when numerical precision is not a primary concern. Moreover, in some simulators, such as Brian, the activation of additional performance options can increase simulation speed dramatically without affecting numerical precision.

Which numerical precision is necessary to conduct a particular study strongly depends on the questions asked. On the one hand, high numerical precision is almost always desirable because it reduces the risk of emerging systematic errors and artifacts in the analyzed system. On the other hand, the higher associated computational cost of high-precision simulations, can render certain types of studies infeasible. The final compromise between simulation speed and simulation precision has to be taken with care and with respect to the exact nature of the scientific questions addressed.

Most network simulations can be sped up even further through parallelism. In particular we compared the run times of the Vogels-Abbott benchmark simulation using NEST, Neuron and Auryn when run on a single machine with 16 cores or a small cluster of four such machines. While parallelization led to increased speed in most cases it was nevertheless difficult to speed up network simulations beyond a tenth of real-time. Since this constitutes a severe restriction in plasticity studies, we analyzed the scaling behavior of Auryn more deeply from which we concluded that this limitation cannot be simply levitated by using more computers.

Specifically we found that in the realm of medium-sized network models with plasticity strong scaling ends for a relatively low number of cores. The origin of this saturation was two-fold: First, network simulations are limited by communication delays when simulated in a distributed fashion on a cluster. Second, larger and more computationally costly simulations additionally suffer from the break-down of strong scaling in each process when high numbers of cores per machine are used. In particular we observed a break-down in scaling behavior in distributed simulations using 48 or 64 out of 64 available physical cores. This effect could be due to the fact that in the latter case no dedicated core is available for the operating system. However, we observed similar saturation effects when using only 48 cores. This seems to suggest that another limiting mechanism is responsible. It is tempting to speculate that it is linked to bandwidth limits in shared memory access on a local machine.

To gain a deeper insight into which parts of a typical network simulation contribute most to the break-down of strong scaling, we performed multiple profiling studies of the parallel simulations in Auryn. This study revealed that while the pure numerical integration of the neuronal differential equations scaled close to linearly in most cases, scaling of spike propagation was generally sub-linear and could become substantial for large numbers of parallel processes. Spike propagation is a memory intensive process because it generally requires to iterate over large fractions of the synaptic weight matrix in a quasi random order due to the stochastic spiking of neurons. These findings therefore seem to support the idea that memory bandwidth limits are indeed the cause behind the breakdown of strong scaling. It will be an interesting avenue for future studies to directly verify this hypothesis in a detailed memory profiling analysis.

Taken together it seems as if the potential for further increase in simulation speed of medium-sized spiking network models on standard hardware is exhausted. Even if it was not for the break-down of strong scaling at the per-process level, strong scaling would still end in distributed simulations at around one tenth of real-time due to communication delays between processes. Therefore, large clusters can only be advantageous if they have extremely low latency communication capabilities. With 10 Gigabit Ethernet becoming increasingly available, a decrease of communication latencies by a factor of 5–10 seems realistic, before yet another performance threshold is reached. At this point a continuation of our study on such machines and on super computers with dedicated low latency communication hardware would be particularly insightful.

With the restrictions at hand it is currently difficult to speed up typical simulations of recurrent networks much further than real-time. In particular this means that a simulation of one day of biological time takes at least several hours to complete. To circumvent performance limitations in simulations of spiking neural networks, GPUs have recently received increasing attention as an inexpensive and massively parallel alternative to distributed simulations (Yudanov et al., 2010; Richert et al., 2011; Brette and Goodman, 2012; Hoang et al., 2013). As for now, it seems as if these approaches are experiencing similar difficulties (Brette and Goodman, 2012) as the ones encountered for the path taken in this manuscript. While real-time simulations are feasible, at present it is not clear if a further decrease of simulation times of networks with realistic plasticity rules is possible.

The evident lack of options to increase simulation speed for large-*time*-scale studies on learning and synaptic plasticity calls for novel ideas of how to approach this type of problem. Noteworthy are approaches addressing neural simulation at the hardware level (Furber and Temple, 2007; Schemmel et al., 2010). Although most of these projects aim at achieving large scale simulations (neuron numbers comparable to the human brain) in real-time, they might also be a good fit for much smaller network configurations where they could provide a significant speed-up. Regardless of which solutions one considers, it would be inevitable that the system brings the necessary flexibility to support a large variety of synaptic plasticity rules ideally without a notable impairment of the overall performance. Finally, it remains an open question if such specialized modeling hardware can ultimately be made available for theory labs worldwide.

In summary we have shown that real-time simulations of plastic networks of point neurons are achievable with appropriate and highly optimized software. However, at the same time increasing simulation speed beyond 10× faster than real-time is challenging due to limitations in the inter-process communications.

## Author contributions

Conceived and designed the research: Friedemann Zenke. Performed the simulations: Friedemann Zenke. Analyzed the data: Friedemann Zenke. Wrote the software: Friedemann Zenke. Wrote the paper: Friedemann Zenke and Wulfram Gerstner.

## Funding

This research was supported by the European Community's Seventh Framework Program under grant agreement no. 237955 (FACETS-ITN), 269921 (BrainScales) and the European Research Council under grant agreement no. 268689 (MultiRules).

### Conflict of interest statement

The authors declare that the research was conducted in the absence of any commercial or financial relationships that could be construed as a potential conflict of interest.

## Footnotes

^1^http://www.fzenke.net/auryn

## References

[B1] Ananthanarayanan R. Esser S. K. Simon H. D. Modha D. S. (2009). The Cat is Out of the Bag Cortical Simulations with 109 Neurons 1013 Synapses. New York, NY: ACM

[B2] Anderson J. D. (1995). Computational Fluid Dynamics: the Basics With Applications. New York, NY: McGraw-Hill

[B3] Bi G.-Q. Poo M.-M. (1998). Synaptic modifications in cultured hippocampal neurons: dependence on spike timing, synaptic strength, and postsynaptic cell type. J. Neurosci. 18, 10464–10472 9852584 10.1523/JNEUROSCI.18-24-10464.1998 PMC6793365

[B4] Bi G.-Q. Poo M.-M. (2001). Synaptic modification by correlated activity: Hebb's postulate revisited. Annu. Rev. Neurosci. 24, 139–166 10.1146/annurev.neuro.24.1.139 11283308

[B5] Bliss T. V. P. Lømo T. (1973). Long-lasting potentiation of synaptic transmission in the dentate area of the anaesthetized rabbit following stimulation of the perforant path. J. Physiol. 232, 331–356 4727084 10.1113/jphysiol.1973.sp010273 PMC1350458

[B6] Brette R. Gerstner W. (2005). Adaptive exponential integrate-and-fire model as an effective description of neuronal activity. J. Neurophysiol. 94, 3637–3642 10.1152/jn.00686.2005 16014787

[B7] Brette R. Goodman D. F. M. (2011). Vectorized algorithms for spiking neural network simulation. Neural. Comput. 23, 1503–1535 10.1162/NECO-a-00123 21395437

[B8] Brette R. Goodman D. F. M. (2012). Simulating spiking neural networks on GPU. Network 23, 167–182 23067314 10.3109/0954898X.2012.730170

[B9] Brette R. Rudolph M. Carnevale T. Hines M. Beeman D. Bower J. (2007). Simulation of networks of spiking neurons: a review of tools and strategies. Front. Comput. Neurosci. 23:349–398 10.1007/s10827-007-0038-6 17629781 PMC2638500

[B10] Brunel N. (2000). Dynamics of sparsely connected networks of excitatory and inhibitory spiking neurons. J. Comput. Neurosci. 8, 183–208 10.1023/A:1008925309027 10809012

[B11] Carnevale N. T. Hines M. L. (2006). The Neuron Book. Cambridge: Cambridge University Press 10.1017/CBO9780511541612

[B12] Davison A. P. Brüderle D. Eppler J. Kremkow J. Muller E. Pecevski D. (2009). PyNN: a common interface for neuronal network simulators. Front. Neuroinform. 2:11 10.3389/neuro.11.011.2008 19194529 PMC2634533

[B13] DeFelipe J. Fariñas I. (1992). The pyramidal neuron of the cerebral cortex: morphological and chemical characteristics of the synaptic inputs. Prog. Neurobiol. 39, 563–607 10.1016/0301-0082(92)90015-7 1410442

[B14] Eliasmith C. Stewart T. C. Choo X. Bekolay T. DeWolf T. Tang Y. (2012). A large-scale model of the functioning brain. Science 338, 1202–1205 10.1126/science.1225266 23197532

[B15] Fehlberg D. E. (1970). Klassische runge-kutta-formeln vierter und niedrigerer ordnung mit schrittweiten-kontrolle und ihre anwendung auf wärmeleitungsprobleme. Computing 6, 61–71 10.1007/BF02241732

[B16] Furber S. Temple S. (2007). Neural systems engineering. J. R. Soc. Interface 4, 193–206 10.1098/rsif.2006.0177 17251143 PMC2359843

[B17] Gerstner W. Kistler W. M. (2002a). Mathematical formulations of hebbian learning. Biol. Cybern. 87, 404–415 10.1007/s00422-002-0353-y 12461630

[B18] Gerstner W. Kistler W. M. (2002b). Spiking Neuron Models: Single Neurons, Populations, Plasticity, 1st Edn. Cambridge: Cambridge University Press 10.1017/CBO9780511815706

[B19] Gerstner W. Kistler W. M. Naud R. Paninski L. (2014). Neuronal Dynamics: From Single Neurons to Networks and Models of Cognition. Cambridge: Cambridge University Press 10.1017/CBO9781107447615

[B20] Gewaltig M.-O. Diesmann M. (2007). NEST (NEural simulation tool). Scholarpedia 2, 1430 10.4249/scholarpedia.1430

[B21] Gewaltig M.-O. Morrison A. Plesser H. E. (2012). NEST by example: an introduction to the neural simulation tool NEST, in Computational Systems Neurobiology, ed Le Novère N. (Dordrecht: Springer), 533–558

[B22] Goodman D. Brette R. (2008). Brian: a simulator for spiking neural networks in python. Front. Neuroinform. 2,5 10.3389/neuro.11.005.2008 19115011 PMC2605403

[B23] Graham S. L. Kessler P. B. Mckusick M. K. (1982). Gprof: a call graph execution profiler, in Proceedings of the 1982 SIGPLAN Symposium on Compiler Construction (New York, NY: ACM), 120–126 10.1145/800230.806987

[B24] Gütig R. Aharonov R. Rotter S. Sompolinsky H. (2003). Learning input correlations through nonlinear temporally asymmetric hebbian plasticity. J. Neurosci. 23, 3697–3714 Available online at: http://www.jneurosci.org/content/23/9/3697.short 12736341 10.1523/JNEUROSCI.23-09-03697.2003 PMC6742165

[B25] Hansel D. Mato G. Meunier C. Neltner L. (1998). On numerical simulations of integrate-and-fire neural networks. Neural Comput. 10, 467–483 10.1162/089976698300017845 9472491

[B26] Henker S. Partzsch J. Schüffny R. (2012). Accuracy evaluation of numerical methods used in state-of-the-art simulators for spiking neural networks. J. Comput. Neurosci. 32, 309–326 10.1007/s10827-011-0353-9 21837455

[B27] Hoang R. V. Tanna D. Bray L. C. J. Dascalu S. M. Harris F. C. Jr. (2013). A novel CPU/GPU simulation environment for large-scale biologically realistic neural modeling. Front. Neuroinform. 7:19 10.3389/fninf.2013.00019 24106475 PMC3788332

[B28] Izhikevich E. M. (2003). Simple model of spiking neurons. IEEE Trans. Neural Netw. 14, 1569–1572 10.1109/TNN.2003.820440 18244602

[B29] Izhikevich E. M. Edelman G. M. (2008). Large-scale model of mammalian thalamocortical systems. Proc. Natl. Acad. Sci. U.S.A. 105, 3593–3598 10.1073/pnas.0712231105 18292226 PMC2265160

[B30] Kandel E. R. Markram H. Matthews P. M. Yuste R. Koch C. (2013). Neuroscience thinks big (and collaboratively). Nat. Rev. Neurosci. 14, 659–664 10.1038/nrn3578 23958663

[B31] Kandel E. R. Schwartz J. H. Jessell T. M. (2000). Principles of Neural Science. New York, NY: McGraw-Hill, Health Professions Division

[B32] Koch C. Reid R. C. (2012). Neuroscience: observatories of the mind. Nature 483, 397–398 10.1038/483397a 22437592

[B33] Lang S. Dercksen V. J. Sakmann B. Oberlaender M. (2011). Simulation of signal flow in 3d reconstructions of an anatomically realistic neural network in rat vibrissal cortex. Neural Netw. 24, 998–1011 10.1016/j.neunet.2011.06.013 21775101

[B34] Markram H. (2006). The blue brain project. Nat. Rev. Neurosci. 7, 153–160 10.1038/nrn1848 16429124

[B35] Markram H. Gerstner W. Sjöström P. J. (2011). A history of spike-timing-dependent plasticity. Front. Synaptic Neurosci. 3:4 10.3389/fnsyn.2011.00004 22007168 PMC3187646

[B36] Markram H. Gerstner W. Sjöström P. J. (2012). Spike-timing-dependent plasticity: a comprehensive overview. Front. Synaptic Neurosci. 4:2 10.3389/fnsyn.2012.00002 22807913 PMC3395004

[B37] Markram H. Lübke J. Frotscher M. Sakmann B. (1997). Regulation of synaptic efficacy by coincidence of postsynaptic APs and EPSPs. Science 275, 213–215 10.1126/science.275.5297.213 8985014

[B38] Morrison A. Aertsen A. Diesmann M. (2007). Spike-timing-dependent plasticity in balanced random networks. Neural Comput. 19, 1437–1467 10.1162/neco.2007.19.6.1437 17444756

[B39] Morrison A. Diesmann M. Gerstner W. (2008). Phenomenological models of synaptic plasticity based on spike timing. Biol. Cybern. 98, 459–478 10.1007/s00422-008-0233-1 18491160 PMC2799003

[B40] Morrison A. Mehring C. Geisel T. Aertsen A. D. Diesmann M. (2005). Advancing the boundaries of high-connectivity network simulation with distributed computing. Neural Comput. 17, 1776–1801 10.1162/0899766054026648 15969917

[B41] Nordlie E. Gewaltig M.-O. Plesser H. E. (2009). Towards reproducible descriptions of neuronal network models. PLoS Comput. Biol. 5:e1000456 10.1371/journal.pcbi.1000456 19662159 PMC2713426

[B42] Pfister J.-P. Gerstner W. (2006). Triplets of spikes in a model of spike timing-dependent plasticity. J. Neurosci. 26, 9673–9682 10.1523/JNEUROSCI.1425-06.2006 16988038 PMC6674434

[B43] Raymond E. S. (2003). The Art of UNIX Programming, 1st Edn. Boston, MA: Addison-Wesley

[B44] Richert M. Nageswaran J. M. Dutt N. Krichmar J. L. (2011). An efficient simulation environment for modeling large-scale cortical processing. Front. Neuroinform. 5:19 10.3389/fninf.2011.00019 22007166 PMC3172707

[B45] Schemmel J. Brüderle D. Grübl A. Hock M. Meier K. Millner S. (2010). A wafer-scale neuromorphic hardware system for large-scale neural modeling, in Proceedings of 2010 IEEE International Symposium on Circuits and Systems (ISCAS) (Paris), 1947–1950 10.1109/ISCAS.2010.5536970

[B46] Sharp T. Furber S. (2013). Correctness and performance of the SpiNNaker architecture, in The 2013 International Joint Conference on Neural Networks (IJCNN) (Dallas, TX), 1–8 10.1109/IJCNN.2013.6706988

[B47] Song S. Miller K. D. Abbott L. F. (2000). Competitive hebbian learning through spike-timing-dependent synaptic plasticity. Nat. Neurosci. 3, 919–926 10.1038/78829 10966623

[B48] Thakur R. Rabenseifner R. Gropp W. (2005). Optimization of collective communication operations in MPICH. Int. J. High Perform. Comput. Appl. 19, 49–66 10.1177/1094342005051521

[B49] van Rossum M. Turrigiano G. (2001). Correlation based learning from spike timing dependent plasticity. Neurocomputing 38–40, 409–415 10.1016/S0925-2312(01)00360-5

[B50] van Vreeswijk C. Sompolinsky H. (1996). Chaos in neuronal networks with balanced excitatory and inhibitory activity. Science 274, 1724–1726 10.1126/science.274.5293.1724 8939866

[B51] Vogels T. P. Abbott L. F. (2005). Signal propagation and logic gating in networks of integrate-and-fire neurons. J. Neurosci. 25, 10786 10.1523/JNEUROSCI.3508-05.2005 16291952 PMC6725859

[B52] Vogels T. P. Sprekeler H. Zenke F. Clopath C. Gerstner W. (2011). Inhibitory plasticity balances excitation and inhibition in sensory pathways and memory networks. Science 334, 1569–1573 10.1126/science.1211095 22075724

[B53] Waldrop M. M. (2012). Computer modelling: brain in a box. Nature 482, 456–458 10.1038/482456a 22358809

[B54] Yudanov D. Shaaban M. Melton R. Reznik L. (2010). GPU-based simulation of spiking neural networks with real-time performance & high accuracy, in The 2010 International Joint Conference on Neural Networks (IJCNN) (Barcelona), 1–8 10.1109/IJCNN.2010.5596334

[B55] Zenke F. Hennequin G. Gerstner W. (2013). Synaptic plasticity in neural networks needs homeostasis with a fast rate detector. PLoS Comput. Biol. 9:e1003330 10.1371/journal.pcbi.1003330 24244138 PMC3828150

[B56] Zhang L. I. Tao H. W. Holt C. E. Harris W. A. Poo M.-M. (1998). A critical window for cooperation and competition among developing retinotectal synapses. Nature 395, 37–44 10.1038/25665 9738497

