# Supplementary material for: Limits to high-speed simulations of spiking neural networks using general-purpose computers
Source: Front Neuroinform. 2014 Sep 11;8:76. doi: 10.3389/fninf.2014.00076 (PMC4160969; doi:10.3389/fninf.2014.00076)
Supplement: Supplementary file 1 [file DataSheet1.PDF]

Frontiers in Neuroinformatics

Research Article
12 August 2014

Supplementary Material: Limits to high-speed simulations of spiking neural networks using generic computers

Friedemann Zenke [1,2,*] and Wulfram Gerstner [1,2]

[1] *School of Life Sciences, Brain Mind Institute, Ecole Polytechnique Federale de Lausanne, Switzerland*
[2] *School of Computer and Communication Sciences, Ecole Polytechnique Federale de Lausanne, Switzerland*

Correspondence*:
Friedemann Zenke
School of Life Sciences, Brain Mind Institute, Ecole Polytechnique Federale de Lausanne, 1015 Lausanne EPFL,Switzerland, friedemann.zenke@epfl.ch

S1 SUPPLEMENTARY FIGURES

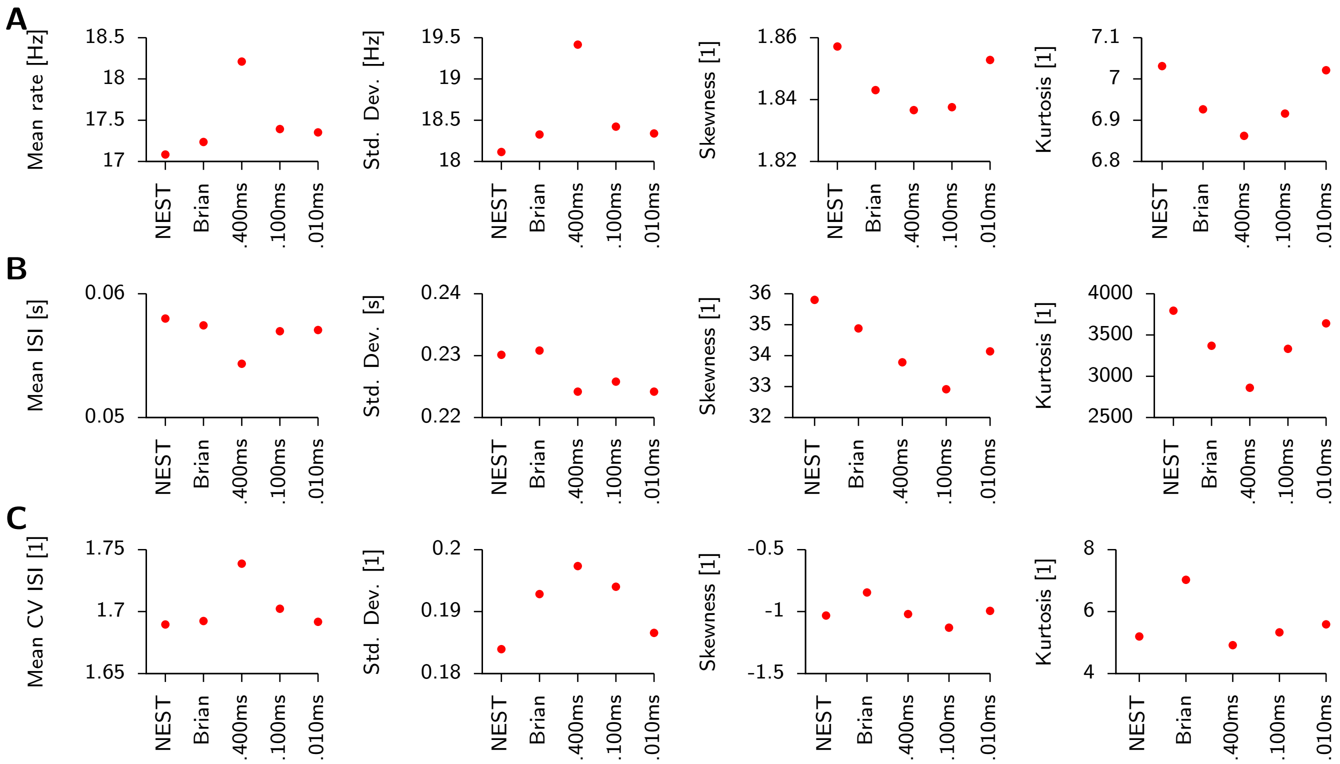

Figure 1. Statistics of rate, ISI and CV ISI distributions in the Vogels-Abbott benchmark. (A-C) Here we plot the mean, standard deviation, skewness and Kurtosis for the data shown in Figure 3 in the main manuscript. All plots show 5 different values: NEST and Brian are the values obtained from the respective reference simulators. While the other values correspond to results for different integration time steps obtained using Auryn. (A) Characterization of the firing rate distributions. (B) Same, but for the ISI distribution. (C) Same, but for the CV ISI distribution.

S2 SUPPLEMENTARY TABLES

In the following we summarize the details and parameters of the networks models used in the main manuscript in tabular form according to **Nordlie et al.** (2009).

Note that both 25,000 cell networks used in this study are in essence identical except their connectivity details. The network firing at 3Hz (which comes with and without STDP) has a connectivity of 5%. If however a higher connectivity of 10% is used the rate drops. The 0.3Hz network uses this higher connectivity in conjunction with non-plastic synapses.

S2.1 VOGELS-ABBOTT BENCHMARK

S2.1.1 Tabular parameter overview

Supplementary Table 1. Vogels Abbott Benchmark: Model Details

A	Model Summary
Populations	Three: excitatory, inhibitory, Poisson input
Topology	–
Connectivity	Random sparse connections
Neuron model	Leaky integrate-and-fire, fixed voltage threshold, fixed absolute refractory time (voltage clamp)
Channel models	–
Synapse model	Exponentially decaying AMPA and GABA conductances and δ-conductance pulses (discontinuous jumps)
Plasticity	–
Input	Initial Poisson input to prime/start the network (during first 50ms)
Measurements	Spike activity

B	Populations	
Name	Size	Elements
E	3200	IF neuron
I	800	IF neuron
Ext	200	Poisson neurons

C	Connectivity		
Name	Source	Target	Pattern
EE	E	E	Random sparse P, probability of connection $\epsilon = 0.02$ weight $w = 0.4$, excitatory
EI	I	E	Random sparse ($\epsilon = 0.02$), $w = 5.1$, inhibitory
IE	E	I	Random sparse ($\epsilon = 0.02$), $w = 0.4$, excitatory
II	I	I	Random sparse ($\epsilon = 0.02$), $w = 5.1$, inhibitory
Ext	Ext	E	Random sparse ($\epsilon = 0.01$), $w = 0.4$, excitatory
			For all connections delay $D = 0.8\text{ms}$

D	Neuron and Synapse Model
Name	IF neuron
Type	leaky integrate-and-fire, exponentially decaying conductance input
Sub-threshold dynamics	If not refractory ($t > t^* + \tau_{\text{ref}}$): $\tau^{\text{m}} \frac{d}{dt} U_i = (U^{\text{rest}} - U_i) + g_i^{\text{exc}}(t)(U^{\text{exc}} - U_i) + g_i^{\text{inh}}(t)(U^{\text{inh}} - U_i) + I^{\text{bg}}$ else: $U_i = U_{\text{rest}}$
Exc. cond.	$\frac{d}{dt} g_i^{\text{exc}} = -g_i^{\text{exc}}/\tau^{\text{exc}} + \sum_{j \in \text{exc}} w_{ij} S_j(t)$
Inh. cond.	$\frac{d}{dt} g_i^{\text{inh}} = -g_i^{\text{inh}}/\tau^{\text{inh}} + \sum_{j \in \text{inh}} w_{ij} S_j(t)$
Spiking	If $U_i > \Theta \quad \wedge \quad t > t^* + \tau_{\text{ref}}$ set $t^* = t$

E	Input
Type	Description
Poisson neurons	Fixed homogenous rate $\nu_{\text{ext}} = 10$

F	Measurements
	Spike activity for raster plots. Run time of simulation (excluding time for setup and saving data).

Supplementary Table 2. Vogels Abbott Benchmark: Model Parameters

G	Model parameter summary	
Symbol	Value	Description
τ^{m}	20ms	Membrane time constant
U^{rest}	-60mV	Resting potential
U^{exc}	0mV	Reversal potential
U^{inh}	-80mV	Inhibitory reversal potential
Θ	-50mV	Spiking threshold
τ_{ref}	5ms	Refractory period
τ^{exc}	5ms	Excitatory conductance time constant
τ^{inh}	10ms	Inhibitory conductance time constant

S2.1.2 Brian simulation code

```
#!/usr/bin/python
from brian import *
from scipy.io import mmread
from pyNN.utility import Timer
from brian.globalprefs import *

###########################################
# Program switches
# #########################################

optimized_code = True
record_spikes  = True

###########################################
# Brian optimizations
# ##########################################

if optimized_code:
     # set_global_preferences(useweave_linear_diffeq=True) # made the
        code slower
     set_global_preferences(useweave=True)
     # set_global_preferences(openmp=True)
     # experimental functions
     set_global_preferences(usecodegen=True)
     # set_global_preferences(usecodegenweave=True)
     # set_global_preferences(usecodegenreset=True)
     # set_global_preferences(usecodegenthreshold=True)
     # set_global_preferences(usecodegenpropagate=True)


###########################################
# Defining network model parameters
# ##########################################

simtime = 60*second # Simulation time

NE = 3200           # Number of excitatory cells
NI = NE/4           # Number of inhibitory cells

w = 1.*nS             # Basic weight unit
tau_ampa = 5.0*ms     # Glutamatergic synaptic time constant
tau_gaba = 10.0*ms    # GABAergic synaptic time constant


# ##########################################
# Neuron model
# ##########################################

gl = 10.0*nsiemens    # Leak conductance
el = -60*mV           # Resting potential
er = -80*mV           # Inhibitory reversal potential
vt = -50.*mV          # Spiking threshold
```

```
memc = 200.0*pfarad  # Membrane capacitance
bgcurrent = 200*pA   # External current

eqs_neurons='''
dv/dt=(-gl*(v-el)-(g_ampa*w*v+g_gaba*(v-er)*w)+bgcurrent)/memc : volt
dg_ampa/dt = -g_ampa/tau_ampa : 1
dg_gaba/dt = -g_gaba/tau_gaba : 1
'''

# ###########################################
# Initialize neuron group
# ###########################################

neurons=NeuronGroup(NE+NI,model=eqs_neurons,threshold=vt,reset=el,
    refractory=5*ms)
neurons.v = er
Pe=neurons.subgroup(NE)
Pi=neurons.subgroup(NI)

# poisson = PoissonGroup(20,rates=lambda t:(t<50*ms)*100*Hz)

# ###########################################
# Connecting the network
# ###########################################

con_ee = Connection(Pe,Pe,'g_ampa')
con_ei = Connection(Pe,Pi,'g_ampa')
con_ie = Connection(Pi,Pe,'g_gaba')
con_ii = Connection(Pi,Pi,'g_gaba')

scale = 10 # scaling factor between weight-scale in file and in
    simulation
con_ee.connect(Pe,Pe,scale*array(mmread('../wmat/ee.wmat').todense(),
    dtype=float))
con_ei.connect(Pe,Pi,scale*array(mmread('../wmat/ei.wmat').todense(),
    dtype=float))
con_ie.connect(Pi,Pe,scale*array(mmread('../wmat/ie.wmat').todense(),
    dtype=float))
con_ii.connect(Pi,Pi,scale*array(mmread('../wmat/ii.wmat').todense(),
    dtype=float))

# ###########################################
# Setting up monitors
# ###########################################

if record_spikes:
    sm = FileSpikeMonitor(Pe,"coba.spikes",record=True)

# ###########################################
# Run without plasticity
# ###########################################

print("Running ...")
```

```
timer = Timer()
timer.start()
run(simtime)
simCPUTime = timer.diff()

print "Simulation time        : %g s" % simCPUTime

if record_spikes:
    raster_plot()
    show()

f = open("timefile.dat",'w')
f.write("%f"%simCPUTime)
f.close()
```

S2.1.3 pyNEST simulation code

```
#!/usr/bin/python
###########################################
# Vogels Abbott benchmark in pyNEST for
# predefined connectivity.
#
# Friedemann Zenke, 2014, fzenke.net
# #########################################

from pyNN.utility import get_script_args, Timer
# pyNN we use for wall clock time measurement
from scipy.io import mmread
import numpy
import nest

###########################################
# Program switches
# #########################################

print_time      = False
euler_integr    = True
record_spikes   = True

###########################################
# Parameters
# #########################################

N_E = 3200
N_I = 800
N_neurons = N_E+N_I

delay=0.8    # ms
J_E = 4.0    # nS
J_I = -51.0  # nS
simtime    = 60*1000.   # [ms]

N_rec = N_E    # Number of neurons to record from
```

```
                # if record_spikes is enabled


#############################################
# Setting parameters of the NEST kernel
# #############################################

nest.SetKernelStatus({"print_time": print_time,
                      "local_num_threads": 1,
                      "overwrite_files": True})


#############################################
# Setting up neuron model
# ###########################################

synapse_model="static_synapse"

if euler_integr:
    neuron_model = "iaf_cond_exp_euler"
else:
    neuron_model = "iaf_cond_exp"

nest.SetDefaults(synapse_model,
        {"delay" : delay,
         "min_delay" : delay,
         "max_delay" : delay } )

nest.SetDefaults(neuron_model,
                 {"E_L": -60.0,
                  "V_th": -50.0,
                  "V_reset": -60.0,
                  "t_ref": 5.0,
                  "E_ex": 0.0,
                  "E_in": -80.0,
                  "C_m" : 200.0,
                  "g_L" : 10.0,
                  "I_e" : 200.0,
                  "tau_syn_ex" : 5.0,
                  "tau_syn_in" : 10.0 })

nodes    = nest.Create(neuron_model,N_neurons)

nodes_E= nodes[:N_E]
nodes_I= nodes[N_E:]

#############################################
# Loading weight matrices
# ###########################################

A=mmread('../wmat/ee.wmat')
rows,cols = A.nonzero()
```

```
nest.Connect(rows+1, cols+1, params = J_E, delay = delay, model="
    static_synapse")

A=mmread('../wmat/ei.wmat')
rows,cols = A.nonzero()
nest.Connect(rows+1, cols+N_E+1, params = J_E, delay = delay, model="
    static_synapse")

A=mmread('../wmat/ie.wmat')
rows,cols = A.nonzero()
nest.Connect(rows+N_E+1, cols+1, params = J_I, delay = delay, model="
    static_synapse")

A=mmread('../wmat/ii.wmat')
rows,cols = A.nonzero()
nest.Connect(rows+N_E+1, cols+N_E+1, params = J_I, delay = delay,
    model="static_synapse")

if record_spikes:
    spikes=nest.Create("spike_detector",1,
                       [{"label": "va-py-ex", "to_file": True}])
    spikes_E=spikes[:1]
    nest.ConvergentConnect(nodes_E[:N_rec],spikes_E)


###########################################
# Simulation and time measurement
# #########################################

timer = Timer()

timer.start()

nest.Simulate(simtime)

runtime = timer.diff()

print "Runtime %f"%runtime

f = open("tf/timefile%d.dat"%nest.Rank(),'w')
f.write("%f\n"%runtime)
f.close()
```

S2.2 BRUNEL NETWORK

Supplementary Table 3. Brunel Balanced Network: Model Details

A	Model Summary
Populations	Three: excitatory, inhibitory, independent Poisson input
Topology	–
Connectivity	Random convergent connections
Neuron model	Leaky integrate-and-fire, fixed voltage threshold, fixed absolute refractory time (voltage clamp)
Channel models	–
Synapse model	δ-current pulses (discontinuous voltage jumps)
Plasticity	–
Input	Independent Poisson input
Measurements	Spike activity

B	Populations	
Name	Size	Elements
E	8000	IF neuron
I	2000	IF neuron
Ext		Poisson generator

C	Connectivity		
Name	Source	Target	Pattern
EE	E	E	Random convergent ($C_\mathrm{E} \to 1$), $w = 0.1\mathrm{mV}$
EI	I	E	Random convergent ($C_\mathrm{E} \to 1$), $w = -0.5\mathrm{mV}$
IE	E	I	Random convergent ($C_\mathrm{I} \to 1$), $w = 0.1\mathrm{mV}$
II	I	I	Random convergent ($C_\mathrm{I} \to 1$), $w = -0.5\mathrm{mV}$
			For all above: $C_\mathrm{E} = 800$, $C_\mathrm{I} = 200$ Delay $D = 0.8\mathrm{ms}$

D	Neuron and Synapse Model
Name	IF neuron
Type	leaky integrate-and-fire, exponentially decaying conductance input
Sub-threshold dynamics	If not refractory ($t > t^* + \tau_\mathrm{ref}$): $\tau^\mathrm{m} \frac{d}{dt} U_i = (U^\mathrm{rest} - U_i) + w_\mathrm{xE} \sum_{j \in \mathrm{E}} S_j(t) + w_\mathrm{xI} \sum_{j \in \mathrm{I}} S_j(t)$ else: $U_i = U_\mathrm{rest}$
Spiking	If $U_i > \Theta \quad \wedge \quad t > t^* + \tau_\mathrm{ref}$ set $t^* = t$
STDP (only in simulations with plasticity)	$\frac{d}{dt} w(t) = \lambda \left(1 - \frac{w(t)}{w_\mathrm{max}}\right) z_j^+(t) S_i(t) - \lambda \alpha \frac{w(t)}{w_\mathrm{max}} z_i^-(t) S_j(t)$ if $w_{ij} < 0$ then $w_{ij} \to 0$ if $w_{ij} > w_\mathrm{max}$ then $w_{ij} \to w_\mathrm{max}$
Synaptic traces	$\frac{dz_n^x}{dt} = -z_n^x/\tau^x + S_n(t)$

E	Input
Type	Description
Poisson neurons	Fixed homogenous rate $\nu_\mathrm{ext} = 10$

F	Measurements
	Spike activity for raster plots. Run time of simulation (excluding time for setup and saving data).

Supplementary Table 4. Brunel Balanced Network: Model Parameters

G	Model parameter summary	
Symbol	Value	Description
τ^{m}	20ms	Membrane time constant
U^{rest}	-60mV	Resting potential
U^{exc}	0mV	Reversal potential
U^{inh}	-80mV	Inhibitory reversal potential
Θ	-50mV	Spiking threshold
τ_{ref}	2ms	Refractory period
Plasticity parameters		
λ	1×10^{-9}	Learning rate
α	2.02	Relative strength of LTD
w_{max}	0.3mV	Maximum weight
$\tau^{+} = \tau^{-}$	20ms	STDP time constant

S2.3 PLASTIC 25,000 CELL NETWORK AT 3HZ

Supplementary Table 5. 25,000 cell network: Model Details

A	Model Summary
Populations	Three: excitatory, inhibitory, Poisson input
Topology	–
Connectivity	Random sparse connections
Neuron model	Leaky integrate-and-fire, moving voltage threshold, absolute and relative refractory time
Channel models	–
Synapse model	Exponentially decaying AMPA, GABA and a slow NMDA conductances. Synaptic input produces δ-conductance pulses (discontinuous jumps) in AMPA and GABA conductances.
Plasticity	–
Input	Poisson input from a population of input units
Measurements	Spike activity

B	Populations	
Name	Size	Elements
E	20,000	IF neuron
I	5,000	IF neuron
Ext	2,500	Poisson neurons

C	Connectivity		
Name	Source	Target	Pattern
EE	E	E	Random sparse, $w = 0.16$, excitatory
EI	I	E	Random sparse, $w = 1.0$, inhibitory
IE	E	I	Random sparse, $w = 0.16$, excitatory
II	I	I	Random sparse, $w = 1.0$, inhibitory
Ext	Ext	E	Random sparse, $w = 0.16$, excitatory
			For all connections: delay $D = 0.8\text{ms}$, probability of connection $\epsilon = 0.05$

D	Neuron and Synapse Model
Name	IF neuron
Type	leaky integrate-and-fire, exponentially decaying conductance input from AMPA and GABA conductances plus a slowly rising and NMDA current with 100ms decay time (voltage dependence ignored)
Sub-threshold dynamics	$\tau^{\text{m}} \frac{dU_i}{dt} = (U^{\text{rest}} - U_i) + g_i^{\text{exc}}(t)(U^{\text{exc}} - U_i) + g_i^{\text{inh}}(t)(U^{\text{inh}} - U_i)$
Conductances	
Excitation	$g_i^{\text{exc}}(t) = \alpha g_i^{\text{ampa}}(t) + (1-\alpha) g_i^{\text{nmda}}(t)$ $\frac{dg_i^{\text{ampa}}}{dt} = -g_i^{\text{ampa}}/\tau^{\text{ampa}} + \sum_{j \in \text{exc}} w_{ij} S_j(t)$ $\tau^{\text{nmda}} \frac{d}{dt} g_i^{\text{nmda}} = -g_i^{\text{nmda}} + g_i^{\text{ampa}}$
Inhibition	$\frac{d}{dt} g_i^{\text{inh}} = -g_i^{\text{inh}}/\tau^{\text{gaba}} + \sum_{j \in \text{inh}} w_{ij} S_j(t)$
Threshold dynamics	$\tau^{\text{thr}} \frac{d}{dt} \vartheta_i = \vartheta^{\text{rest}} - \vartheta_i$
Spiking	If $U_i > \vartheta$ then $t^* = t$ and $\vartheta \to +50\text{mV}$ and $U_i \to U^{\text{rest}}$.
Triplet STDP	$\frac{d}{dt} w_{ij} = \eta w_0 \, A^+ \, z_j^+(t) \, z_i^{\text{slow}}(t-\epsilon) \, S_i(t) - \eta w_0 \, A_i^-(t) \, z_i^-(t) \, S_j(t)$ and $A_i^-(t) = \frac{A^+ \tau^+ \tau^{\text{slow}}}{\tau^- \kappa} \bar{\nu}_i(t)^2$ if $w_{ij} < 0$ then $w_{ij} \to 0$ if $w_{ij} > w_{\max}$ then $w_{ij} \to w_{\max}$
Synaptic traces	$\frac{d}{dt} z^x = -z^x/\tau^x + S_n(t)$
Homeostatic rate estimate	$\tau \frac{d}{dt} \bar{\nu} = -\bar{\nu} + S_n(t)$

Supplementary Table 6. 25,000 cell network: Model details (continued) and model parameters

E	Input
Type	Description
Poisson neurons	Fixed homogenous rate $\nu_{\text{ext}} = 2$

F	Measurements
	Spike activity for raster plots. Run time of simulation (excluding time for setup and saving data).

G	Model parameter summary	
τ^{m}	20ms	Membrane time constant
U^{rest}	-60mV	Resting potential
U^{exc}	0mV	Reversal potential
U^{inh}	-80mV	Inhibitory reversal potential
ϑ^{rest}	-50mV	Spiking threshold
τ^{thr}	5ms	Refractory period
τ^{ampa}	5ms	AMPA conductance time constant
τ^{nmda}	100ms	NMDA conductance decay time constant
τ^{gaba}	10ms	Inhibitory conductance time constant
α	0.5	Ratio between AMPA and NMDA conductance
Plasticity parameters		
η	6.25	Relative learning rate
w_0	0.16	Initial synaptic weight
A^+	$6.5 \times 10^{-3}\eta$	Potentiation strength
τ^+	16.8ms	Trace time constant LTP
τ^-	33.7ms	Trace time constant LTD
τ^{slow}	114ms	Long trace time constant LTP
w^{max}	1.0	Maximally allowed weight value
κ	3Hz	Target rate

REFERENCES

Nordlie, E., Gewaltig, M.-O., and Plesser, H. E. (2009), Towards reproducible descriptions of neuronal network models, *PLoS Comput Biol*, 5, 8, e1000456, doi:10.1371/journal.pcbi.1000456
